# Deletion of Neurotrophin Signaling through the Glucocorticoid Receptor Pathway Causes Tau Neuropathology

**DOI:** 10.1038/srep37231

**Published:** 2016-11-16

**Authors:** Margarita Arango-Lievano, Camille Peguet, Matthias Catteau, Marie-Laure Parmentier, Synphen Wu, Moses V Chao, Stephen D. Ginsberg, Freddy Jeanneteau

**Affiliations:** 1 Inserm, U1191, Institute of Functional Genomics, F-34000 Montpellier, France; 2 CNRS, UMR-5203, F-34000 Montpellier, France; 3 Université de Montpellier, F-34000 Montpellier, France; 4 Skirball Institute of biomolecular medicine, New York University Langone Medical Center, New York, NY 10016, USA; 5 Center for Dementia Research, Nathan Kline Institute, Departments of Psychiatry, Neuroscience & Physiology, New York University Langone Medical Center, Orangeburg, NY 10962, USA

## Abstract

Glucocorticoid resistance is a risk factor for Alzheimer’s disease (AD). Molecular and cellular mechanisms of glucocorticoid resistance in the brain have remained unknown and are potential therapeutic targets. Phosphorylation of glucocorticoid receptors (GR) by brain-derived neurotrophic factor (BDNF) signaling integrates both pathways for remodeling synaptic structure and plasticity. The goal of this study is to test the role of the BDNF-dependent pathway on glucocorticoid signaling in a mouse model of glucocorticoid resistance. We report that deletion of GR phosphorylation at BDNF-responding sites and downstream signaling via the MAPK-phosphatase DUSP1 triggers tau phosphorylation and dendritic spine atrophy in mouse cortex. In human cortex, DUSP1 protein expression correlates with tau phosphorylation, synaptic defects and cognitive decline in subjects diagnosed with AD. These findings provide evidence for a causal role of BDNF-dependent GR signaling in tau neuropathology and indicate that DUSP1 is a potential target for therapeutic interventions.

Stress is a major risk factor for promoting or aggravating neuropathological changes within the cortico-limbic system in neuropsychiatric, neurodegenerative and metabolic disorders^1^,^2^,^3^. Circumstantial evidence points towards an excessive secretion of glucocorticoid hormones (cortisol in humans and corticosterone in rodents) in these disease states^4^. Normally, glucocorticoids are secreted in synchrony with circadian rhythms and in response to novelty and stress, virtually accessing all cells in the organism to prepare physiological adaptive responses to the changing environment^5^. In the brain, glucocorticoids can either facilitate or dampen the structure and function of circuits in the cortico-limbic system involved in reward learning and aversion learning^6^,^7^,^8^. The response outcome to glucocorticoids, beneficial or deleterious for neurons, depends on the age, sex, dose, duration and context at exposure^9^.

Neurobiological correlates of glucocorticoid plasticity consist in epigenetic modifications, mitochondrial function and cytoskeletal remodeling based on changes in phosphorylation of signaling and structural proteins^10^,^11^. Molecular substrates of glucocorticoid plasticity include, but are not limited to, phosphorylation of cytoskeletal-associated proteins in the LIMK1/cofilin/actin pathway and the tau/MAP/microtubule pathway^12^,^13^. Glucocorticoids augment tau phosphorylation at several epitopes associated with cytoskeletal pathology, synaptic loss, neuronal atrophy and cognitive impairment^13^. Tau hyperphosphorylation is obligatory for glucocorticoids to accelerate stress-induced neuropathology^14^. Stress and glucocorticoids change the expression and activity of tau kinases, suggesting that neuropathology induced by cumulative stress occurs via abnormal phosphorylation waves and gene transcription^13^,^15^.

The primary factor of vulnerability to cumulative stress is the acquired resistance of cells to the actions of glucocorticoids^16^,^17^. Partial signaling via glucocorticoid receptors (GR) is postulated in several disorders featuring chronic inflammation including AD^17^,^18^,^19^,^20^. A negative consequence of GR resistance is partial suppression of the secretion of stress hormones by the hypothalamic-pituitary-adrenal (HPA) axis resulting in elevated circulating concentrations of these hormones for prolonged periods of time^21^. Currently, molecular and cellular mechanisms of GR resistance in neurons are not well understood.

GR is a central pivot of glucocorticoid responses at the intersection of multiple signaling pathways for stress/depression systems, cognitive functions, and synaptic plasticity, notably the growth factor signaling pathways^22^,^23^. Behavioral studies indicated that glucocorticoid responses on cortico-limbic circuits are altered by brain-derived neurotrophic factor (BDNF) signaling in the brain^7^,^24^,^25^. Previously, we characterized roles of BDNF on the responses of GR signaling in the hypothalamus and cortex^26^,^27^. Notably, proteomic and transcriptomic approaches revealed unique post-translational modifications on GR and specific genes regulated by stimulation of cortical neurons with glucocorticoids alone or when paired with BDNF^27^. Phosphorylation of GR at BDNF-responding sites is linked to dendritic spine growth, and sensitive to chronic stress and antidepressant treatment^28^. Permissive effects of BDNF signaling on glucocorticoid transcriptional responses are expected to rely on the phosphorylation of GR and interaction with co-regulators (Fig. 1A). We propose a novel mechanism to explain how glucocorticoid activities can change from beneficial to detrimental. It relies on the context at exposure, if BDNF signaling is ‘ON’ or ‘OFF’ at the time of glucocorticoid stimulation to produce a unique response (for review see ref. 23).

One strategy to demonstrate permissivity is to disrupt one signaling hub that integrates both glucocorticoid and BDNF pathways. This study explored GR phosphorylation as such a hub in the cortex of a mouse model featuring disrupted glucocorticoid circadian rhythms (Supplementary Figure S1).

## Results

### GR phosphorylation at BDNF-responding sites in a model of chronic treatment with corticosterone

**Figure 1:**
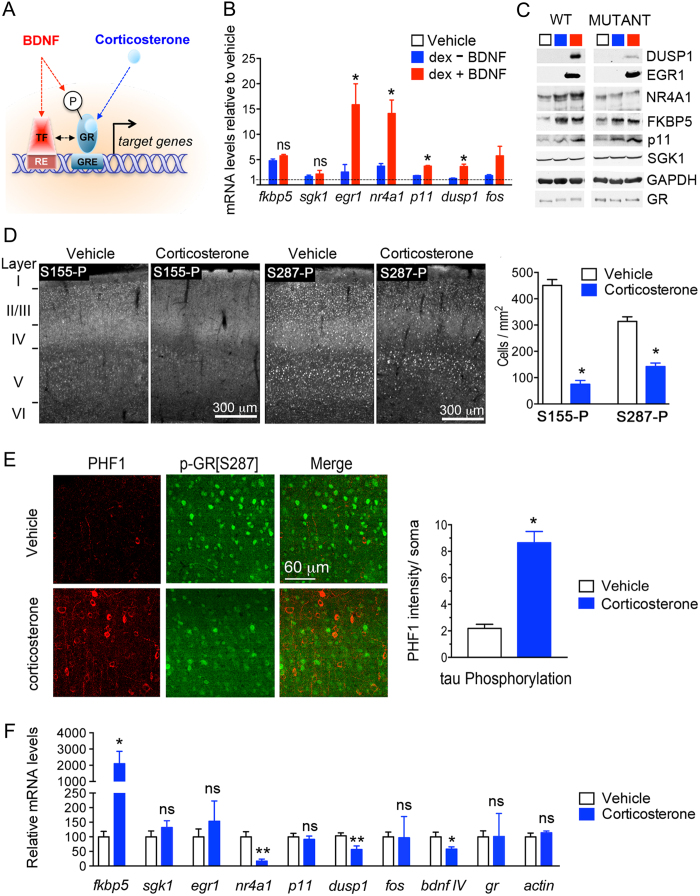
Chronic exposure to corticosterone decreases GR phosphorylation at BDNF-responding sites, DUSP1 expression and increases tau phosphorylation in mouse cortex. (**A**) Model describing permissive effects of BDNF on corticosterone responses notably via GR phosphorylation. TF: transcription factor, RE: responsive element, GRE: glucocorticoid-RE, P: phosphorylation. (**B**) Q-PCR of GR-regulated genes in primary cortical neurons stimulated for 3 hrs with 1 μM dex, 50 ng/ml BDNF or both. Mean ± SEM of at least 3 independent experiments, unpaired t-test *P < 0.05. (**C**) Effect of acute stimulation of primary cortical neurons for 3 hrs with 1 μM dex, 50 ng/ml BDNF or both on protein expression of GR-regulated genes. Neurons were infected with lentivirus prior stimulation for expressing wildtype or the mutant GR[S155A/S287A] that can no longer be phosphorylated by BDNF. (**D**) Phosphorylation of GR at BDNF-responding sites [S155 and S287] is reduced in cortex of mice treated daily with 15 mg/kg corticosterone for 10 days. Mean ± SEM of N = 11 mice/group, unpaired t-test *P < 0.0001. (**E**) Somatic phosphorylation of tau (PHF1) in cortex of mice exposed to chronic treatment with corticosterone. Mean ± SEM, unpaired t-test *P < 0.0001, N = 11 mice/group. (**F**) Expression of corticosterone-regulated genes in cortex measured by Q-PCR. Mean ± SEM of N = 3–7 vehicle groups and 3–6 corticosterone groups, unpaired t-test *P < 0.05, **P < 0.01.

Regulation of neuronal gene expression by acute stimulation of GR with glucocorticoids, natural (corticosterone) or synthetic (dexamethasone), is under the influence of BDNF signaling^27^. For instance, induction of *DUSP1* (Dual Specificity Phosphatase-1*/mkp1*), *EGR1* (Early Growth Response-1), *NR4A1* (Nuclear Receptor subfamily-4 Group-A member-1/nur77/NGF1A), *P11* (S100A10) and *FOS* (FBJ Osteosarcoma viral Oncogene homolog/AP1) in primary cortical neurons responded to co-stimulation with BDNF and dexamethasone (Fig. 1B), but only *DUSP1* and *NR4A1* required GR phosphorylation at BDNF responsive sites to synthesize the protein product (Fig. 1C). In contrast, chronic stimulation with glucocorticoids is likely to reduce the responsiveness of GR signaling in cortical neurons by interfering with BDNF expression in the mouse brain^29^,^30^.

To explore the impact of chronic glucocorticoid treatment on the phosphorylation of GR at BDNF responding sites, mice were injected daily with corticosterone (15 mg/kg, intraperitoneal) for 10 consecutive days beginning at PND21 (Supplementary Figure S1A). Morning injection at the glucocorticoid circadian trough raised blood levels of corticosterone till the evening, returning to normal on the next day (Supplementary Figure S1B). This treatment regimen resulted in behavioral despair in the tail suspension test (Supplementary Figure S1C), deficits of BDNF (Supplementary Figure S1D), synaptic atrophy (Supplementary Figure S1E–G) and decrease of GR phosphorylation at BDNF responding sites (Fig. 1D) paralleled with *de novo* phosphorylation of tau (PHF1 staining, Fig. 1E), within the somatosensory cortex.

Decreased phosphorylation of GR is consistent with altered expression of *dusp1* and *nr4a1* in cortex (Fig. 1F). Other genes *(egr1, p11 and fos)* responding to acute co-stimulation with BDNF and dexamethasone are not changed by chronic corticosterone exposure. Therefore, despite the high circulating levels of corticosterone, GR downstream signaling was minimized as typically featured in physiological states of glucocorticoid resistance.

### Significance of a disruption of GR phosphorylation at BDNF responding sites *in vivo*

**Figure 2:**
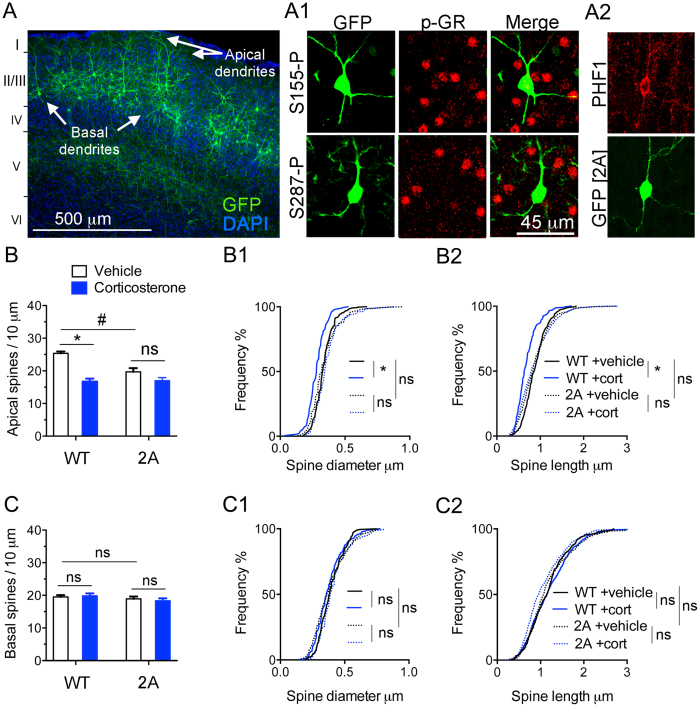
Disruption of GR phosphorylation at BDNF-sensitive sites *in vivo* causes tau phosphorylation and dendritic spine loss. Mouse cortex electroporated with a molecular replacement vector (**A**) to substitute endogenous GR with the GR-2A mutant that no longer can be phosphorylated at the S155 and S287 sites (A1). (A2) Effect of GR-2A on tau phosphorylation detected with PHF1 immunoreactivity. (**B**) Spine density (Mean ± SEM) at apical tufts of LII/III excitatory neurons in somatosensory cortex. 2-way ANOVA for the effect of corticosterone compared to vehicle F(1,20) = 38.12, effect of GR-2A compared to GR-WT F(1,20) = 8.75, effect of interaction F(1,20) = 10.52 posthoc Tukey’s test ^#^P = 0.0015, *P < 0.0001, N = 6 mice/group. Cumulative distribution of apical spines according to their diameter (B1) and length (B2). 2-way ANOVA, post-hoc Tukey’s test *P < 0.02, N > 200 spines/ group. (**C**) Spine density (Mean ± SEM) at basal dendrites. 2-way ANOVA for the effect of corticosterone F(1,16) = 0.022, effect of GR-2A F(1,16) = 2.238, effect of interaction F(1,16) = 0.38, N = 5 mice/group. Cumulative distribution of apical spines according to their diameter (C1) and length (C2). 2-way ANOVA, post-hoc Tukey’s test, N > 200 spines/group.

**Figure 3:**
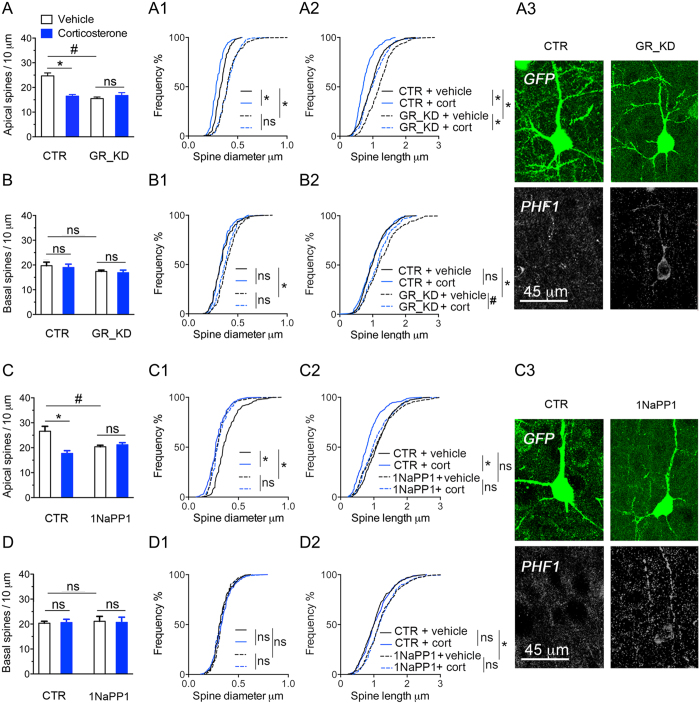
Loss-of-function of GR and TrkB *in vivo* causes tau phosphorylation and dendritic spine loss. (**A**) Spine density (Mean ± SEM) at apical tufts of LII/III excitatory neurons in somatosensory cortex. 2-way ANOVA for the effect of corticosterone compared to vehicle F(1,16) = 10.3, effect of GR knockdown (GR_KD) compared to shRNA control (CTR) F(1,16) = 15.15, effect of interaction F(1,16) = 18.76 posthoc Tukey’s test ^#^P = 0.0001, *P = 0.0004, N = 5 mice/group. Cumulative distribution of apical spines according to their diameter (A1) and length (A2). 2-way ANOVA, post-hoc Tukey’s test *P < 0.0001, N > 200 spines/ group of 5 mice each. (A3) Effect of GR_KD on tau phosphorylation detected with PHF1 immunoreactivity. (**B**) Spine density (Mean ± SEM) at basal dendrites. 2-way ANOVA for the effect of corticosterone F(1,16) = 0.28, effect of GR_KD F(1,16) = 3.73, effect of interaction F(1,16) = 0.007, posthoc Tukey’s test P > 0.05, N = 5 mice/group. Cumulative distribution of basal spines according to their diameter (B1) and length (B2). 2-way ANOVA, post-hoc Tukey’s test *P < 0.0001, ^#^P < 0.05, N > 200 spines/ group. (**C**) Spine density (Mean ± SEM) at apical tufts of LII/III excitatory neurons in somatosensory cortex. 2-way ANOVA for the effect of corticosterone compared to vehicle F(1,16) = 10.63, effect of 1NaPP1 (TrkB[F669A] inactivation) compared to water control (CTR) F(1,16) = 1.24, effect of interaction F(1,16) = 15.23 posthoc Tukey’s test ^#^P = 0.0129 *P = 0.0006, N = 5 mice/group. Cumulative distribution of apical spines according to their diameter (C1) and length (C2). 2-way ANOVA, post-hoc Tukey’s test *P < 0.0001, N > 200 spines/ group. (C3) Effect of TrkB deactivation with 1NaPP1 on tau phosphorylation detected with PHF1 immunoreactivity. (**D**) Spine density (Mean ± SEM) at basal dendrites. 2-way ANOVA for the effect of corticosterone F(1,16) = 0.0028, effect of 1NaPP1 control (CTR) F(1,16) = 0.067, effect of interaction F(1,16) = 0.83, posthoc Tukey’s test P > 0.05, N = 5 mice/group. Cumulative distribution of basal spines according to their diameter (B1) and length (B2). 2-way ANOVA, post-hoc Tukey’s test, *P < 0.0001, N > 200 spines/ group.

To disrupt GR phosphorylation sites *in vivo*, we electroporated the mouse cortex with a DNA construct to knockdown endogenous GR and express the recombinant BDNF-insensitive phospho-deficient GR mutant [2A for S155A/S287A] made resistant to the shRNA. This construct also expresses GFP to permit analysis of dendritic spines in cortical tissue sections. At one month of age, electroporated GFP-positive neurons lacked phospho-isoforms S155 and S287 (Fig. 2A1), and showed staining for PHF1 (Fig. 2A2). Remarkably, expression of GR-2A construct reduced the number of dendritic spines in apical tufts of the layer LII/III excitatory neurons without altering basal dendrites, thereby recapitulating the effects of chronic corticosterone treatment (Fig. 2B,C). The absence of additive effects between GR-2A and chronic corticosterone treatment suggests redundancy in the mechanisms at play. Additionally, expression of GR-2A mutant recapitulated the loss-of-function of GR knockdown on the responses of dendritic spines to corticosterone in LII/III excitatory neurons of somatosensory cortex and PHF1 staining (Fig. 3A,B). This result is not an experimental artifact given that spines from apical tuft dendrites of neurons expressing GR-WT construct (Fig. 2B) or shRNA CTR (Fig. 3A) responded to chronic treatment with corticosterone. Taken together the effects of prolonged exposure to corticosterone on spine number result from a loss-of-function of GR that could be attributable to deficits of BDNF signaling. This is in part corroborated by the reduced levels of BDNF protein found in somatosensory cortex of mice treated with chronic corticosterone (Supplementary Figure S1D).

To mimic deficits of BDNF signaling *in vivo*, we substituted endogenous TrkB with a mutant TrkB-F669A that can be inactivated with 1NaPP1, using a construct expressing a shRNA against TrkB carrying a GFP reporter cassette and a shRNA resistant TrkB-F669A fused to RFP. We previously reported that this manipulation reduces GR phosphorylation in cortical neurons^28^. *In vivo*, 1NaPP1 was supplied at 25 mM in drinking water during chronic corticosterone exposure. We found that 1NaPP1 recapitulated the loss-of-function of GR-2A mutant and GR knockdown on salient responses of dendritic spines to corticosterone (Fig. 3C,D). Additionally, GFP-positive neurons exposed to 1NaPP1 presented with *de novo* PHF1 immunoreactivity (Fig. 3C3). Consequently, excitatory cortical neurons require GR, GR phosphorylation and TrkB signaling to maintain dendritic spines and to respond to corticosterone. This result illustrates functional redundancy caused by the blockade of TrkB signaling and deficiency of GR phosphorylation *in vivo*.

### GR phosphorylation downstream signaling through *Dusp1*

**Figure 4:**
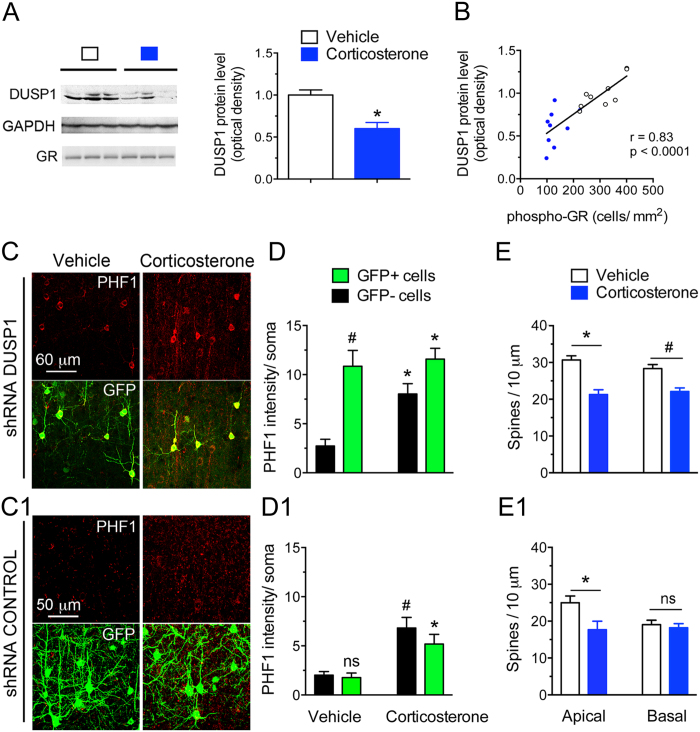
Loss-of-function of the GR phosphorylation-dependent gene *DUSP1* exaggerates the responses of cortical neurons to chronic corticosterone treatment. (**A**) Cortical amounts of DUSP1 protein measured by Western blot analysis. Each lane represents an independent mouse sample. Mean ± SEM normalized to vehicle controls. Unpaired t-test *P = 0.0006, N = 9 mice/group. (**B**) Same data plotted as a function of GR phosphorylation [S287] measured by immuno histochemistry in contralateral cortex. Pearson correlation of data from 9 mice/ group. (**C**) Tau phosphorylation [PHF1] in mouse cortex of mice treated with chronic corticosterone. LII/III pyramidal neurons were electroporated *in utero* with a shRNA against DUSP1 carrying a GFP reporter (**C**) or the shRNA control (C1). (**D**) 2-way ANOVA for the effect of shRNA DUSP1 F(1,20) = 25.1, effect of corticosterone F(1,20) = 6.66, post-hoc Tukey’s test *P < 0.025, ^#^P = 0.001. About 20 cells per brain slices from N = 5 mice for the vehicle group and 7 for corticosterone. (D1) 2-way ANOVA for the effect of shRNA control F(1,24) = 1.423, effect of corticosterone F(1,24) = 26.7, post-hoc Tukey’s test *P = 0.021, ^#^P = 0.002. N = 7 mice for vehicle groups and 6–8 for corticosterone. (**E**) Spine density (Mean ± SEM) at apical and basal dendrites of LII/III pyramidal neurons electroporated with the shRNA DUSP1 in mouse cortex. Unpaired t-test, *P = 0.0003, ^#^P = 0.0012, N = 7 mice/ group. (E1) Spine density (Mean ± SEM) at apical and basal dendrites of LII/III pyramidal neurons electroporated with the shRNA control. Unpaired t-test, *P = 0.038, N = 5–7 mice for the shRNA control and shRNA DUSP1 vehicle-treated groups and 5–6 for corticosterone-treated groups.

Elucidating the signaling pathways downstream of GR phosphorylation that are involved in activity-dependent formation/maintenance of synaptic connections and tau phosphorylation could provide insightful information about neuronal (mal)adaptation to chronic glucocorticoid exposure and diseases featuring hypercortisolemia as a risk factor (e.g. AD, cognitive impairment). For instance, *dusp1* is an immediate early gene which transcription is sensitive to BDNF and glucocorticoids, and required GR phosphorylation, TrkB, ERK1/2 and CREB^20^,^31^,^32^. Cortical levels of DUSP1 and GR phosphorylation are correlated, higher in mice treated with vehicle than chronic corticosterone (Fig. 4A,B).

This prompted us to downregulate experimentally DUSP1 expression in mouse cortex with a validated shRNA vector carrying a GFP reporter (Supplementary Figure S2). DUSP1 knockdown resulted in tau hyperphosphorylation in GFP-positive cortical neurons compared to cells expressing the shRNA control (Fig. 4C,C1). Chronic corticosterone treatment increased PHF1 immunoreactivity in neurons expressing the shRNA against DUSP1 (Fig. 4D) and decreased spine density at apical and basal dendrites (Fig. 4E) more than in cells expressing the shRNA control (Fig. 4D1,E1), suggestive of an aggravated response to corticosterone when *dusp1* expression is low.

### DUSP1 gain-of-function *in vivo*

**Figure 5:**
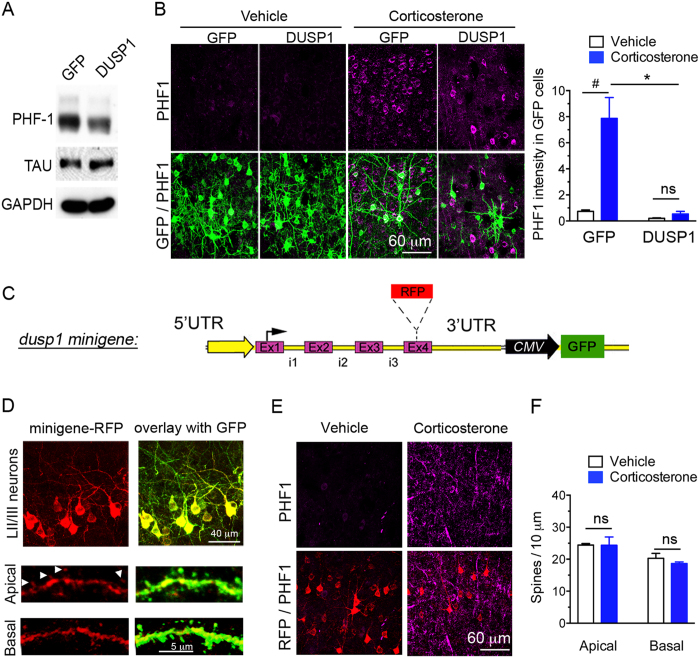
Gain-of-function of DUSP1 reduces tau[PHF1] phosphorylation and prevents the responses of dendritic spines to chronic corticosterone treatment. (**A**) Transduction of DUSP1 in primary cortical neurons (DIV10) reduces tau[PHF1] phosphorylation compared to transduction of GFP. (**B**) PHF1 expression (Mean ± SEM) in cortex electroporated *in utero* to overexpress DUSP1 and GFP in LII/III neurons. 2-way ANOVA for the effect of DUSP1 F(1,20) = 52.67, effect of corticosterone F(1,20) = 47.49, post-hoc Tukey’s test *P < 0.0001, ^#^P < 0.0001, N = 4 GFP and 6 DUSP1 mice in vehicle groups and 6 GFP, 8 DUSP1 mice in CORT groups. (**C**) DUSP1 minigene construct encompassing 5 kilobases of the gene. (**D**) DUSP1-RFP minigene expressed in LII/III pyramidal neurons of mouse cortex localized in dendrites and spines (arrows). (**E**) PHF1 expression weakly expressed in LII/III pyramidal neurons electroporated with the minigene-RFP in mouse cortex of mice treated with chronic corticosterone. (**F**) Spine density (Mean ± SEM) at apical and basal dendrites of LII/III pyramidal neurons electroporated with the minigene DUSP1-RFP in mouse cortex. Unpaired t-test, P > 0.05, N = 4–5 vehicle and 4–5 corticosterone mice/ group.

One question remains if overexpression of *DUSP1* could decrease PHF1 levels in cortical neurons and could prevent dendritic spine loss mediated by prolonged corticosterone exposure. Gain-of-function was achieved with lentivirus in primary cortical neurons and in mouse cortex by *in utero* electroporation. We found that DUSP1 decreased PHF1 levels *in vitro* (Fig. 5A), and *in vivo* DUSP1 overexpressing cells (GFP+) barely exhibited PHF1 immunoreactivity (Fig. 5B). This is not an experimental artifact because cells expressing GFP alone responded to corticosterone by increasing PHF1 immunoreactivity. Importantly, long-term overexpression of DUSP1 reduced neuronal growth, which could introduce bias.

To gain modest expression and at the same time localize DUSP1 *in vivo*, we constructed a minigene encompassing the 5 kilobases of the 4 exons and introns encoding for the protein DUSP1 to which we fused a RFP cassette, plus 1 kilobase of 5′UTR and 1 kilobase of 3′UTR (Fig. 5C). This construct allowed expression of DUSP1-RFP by its own regulatory elements and a constitutive GFP reporter in mouse cortex after *in utero* electroporation (Fig. 5D). DUSP1-RFP was absent from the nucleus of excitatory cortical neurons and localized in axons, dendrites, and spines (Fig. 5D insets). Cells expressing the minigene barely showed PHF1 immunoreactivity (Fig. 5E). Additionally, cells expressing the minigene presented with the same number of dendritic spines whether exposed to a chronic treatment with corticosterone or with vehicle (Fig. 5F). Therefore, gain-of-function of DUSP1 counteracted salient neurochemical defects caused by a chronic treatment with glucocorticoids in mice.

### DUSP1 expression in human brain

**Figure 6:**
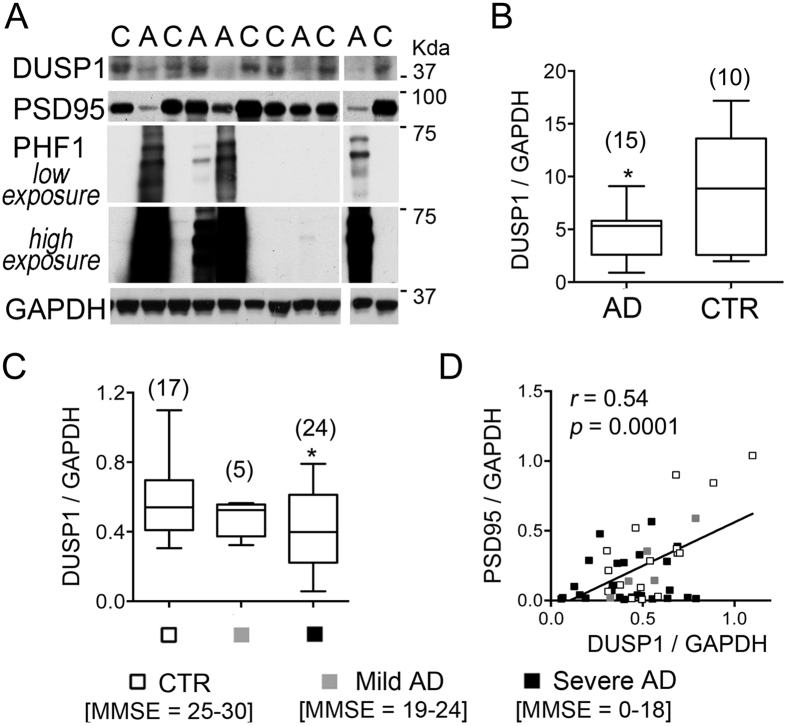
Loss of DUSP1 in the human brain with AD. (**A**) Western blot analysis of protein expression in cortical lysates of human brains (C = healthy controls, A = AD). (**B**) Densitometric analysis of DUSP1 protein levels normalized to GAPDH (Mean ± min to max) in cohort 1 consisting of 10 healthy controls and 15 AD cases not tested for MMSE scores (unpaired t-test *P = 0.02). (**C**) Densitometric analysis of DUSP1 protein levels normalized to GAPDH (Mean ± min to max) in cohort 2 consisting of 17 controls, 29 AD subjects amongst which 5 showed mild cognitive impairment and 24 showed severe cognitive impairment based on MMSE scores. Unpaired t-test CTR versus severe *P = 0.02. (**D**) DUSP1 levels as a function of PSD95 expression. Pearson correlation of data from N = 46 patients, P < 0.0001. Mean ± SEM of PSD95 levels in CTR 0.36 ± 0.078, MILD 0.22 ± 0.08, SEVERE 0.14 ± 0.034, Unpaired t-test CTR versus severe *P = 0.008.

Our *in vitro* and *in vivo* modeling studies prompted us to assess whether DUSP1 can be considered as a potential therapeutic target for disorders featuring hallmarks of glucocorticoid resistance, one of which is hypercortisolemia. High blood levels of glucocorticoids through chronic stress, chronic therapies or mutations of genes involved in the signaling of cortisol are reported to exacerbate tau phosphorylation, synaptic loss and cognitive decline in AD^33^,^34^,^35^. To determine DUSP1 expression in the human AD brain, we analyzed postmortem frontal cortical tissues from individuals with AD and non-demented controls that were matched for age, gender, and postmortem interval (Supplementary Figure S3). First, we extracted total proteins from Broadmann area BA9-10 of 15 cases diagnosed with AD compared to 10 control subjects. Excessive phosphorylation of tau with PHF1 immunoreactivity indicates signs of tau pathology in the tissue samples with AD (Fig. 6A). In these samples, mean levels of DUSP1 protein are significantly lower compared to healthy controls (Fig. 6B).

A second cohort with 29 subjects clinically diagnosed with AD and 17 non-demented controls all with cognitive assessments prior to death using the Mini-Mental State Exam (MMSE) permitted correlations of cortical expression of DUSP1 with an index of cognitive decline. Mean levels of DUSP1 protein are reduced only in the category presenting severe cognitive deficits (Fig. 6C), proportionate to Braak stages (Supplementary Figure S3D). Interestingly, DUSP1 protein levels correlate with that of PSD95 (Fig. 6D), a postsynaptic marker associated with glutamatergic neurotransmission that is downregulated in the AD group (–59.92 ± 9.52% in 24 AD versus 17 CTR, unpaired t-test P = 0.008 and^36^) and in a mouse model of tauopathy^37^. Taken together, these results indicate that DUSP1 protein is downregulated in the human AD brain featuring synaptic pathology, aberrant tau phosphorylation, cognitive decline, and for which hypercortisolemia is a risk factor^38^.

## Discussion

Hypersecretion of glucocorticoids is a risk factor for accelerating neuropathological changes associated with AD in humans and in relevant AD animal models^38^,^39^. In contrast to the continuously elevated blood levels that are characteristic of hypercortisolemia in Cushing’s disease or major depression, hypersecretion of cortisol in AD could be phasic from a low circadian trough to exaggerated concentrations at circadian peaks^40^,^41^. Our study in mice explored the effects of a chronic treatment with corticosterone, which recapitulated such a phasic pattern of circulating corticosterone levels, on the development of chronic insufficiency of GR signaling and related neuropathology.

Evidence for insufficient signaling of GRs despite elevated circulating levels of glucocorticoids is provided by studies in rats either injected chronically with a fragment of the β-amyloid peptide (Aβ25-35) in cerebrovascular milieu^42^ or subjected to chronic restrain stress^43^. In these conditions, nucleo-cytoplasmic shuttling of GR might be impaired and phosphorylation of GR by CDK5 or JNK might be important for controlling the transcriptional response of glucocorticoids. However, these studies did not produce molecular manipulations to elucidate possible mechanisms of GR signaling insufficiency. Herein presented are manipulations targeting GR phosphorylation at physiologically-relevant BDNF-responding sites and downstream signaling to impair responses of cortical neurons to a chronic treatment with glucocorticoids: (1) the deactivation of BDNF signaling through TrkB receptors and (2) the deletion of GR phosphorylation sites. Both manipulations elicited *de novo* phosphorylation of tau and dendritic spine atrophy recapitulating salient effects of chronic corticosterone treatment. Although a floor effect cannot be completely excluded, we interpret deficits of GR phosphorylation as evidence of GR resistance in the absence of additive effects between GR manipulations and cumulative corticosterone exposure, which is translationally impactful for neurodegenerative disorder research as well as neuropsychiatric disorder research.

Among the genes dependent on BDNF and GR phosphorylation, DUSP1 expression is involved in suppressing the occurrence of hyperphosphorylated tau and synaptic loss in mouse cortex in response to a chronic treatment with corticosterone. In human cortex, DUSP1 expression correlates with PHF1 immunoreactivity, PSD95 levels, Braak stage and MMSE scores. Overall, DUSP1 is downregulated in AD brains compared to controls in 2 cohorts. We did not test the expression of GR phosphorylation in postmortem human tissues because GR phosphorylation is short-lived and very sensitive to dephosphorylation *in vitro,* likely making postmortem analysis equivocal^28^. A link between GR phosphorylation at BDNF-responding sites and DUSP1 expression in human brain remains to be demonstrated, ideally by mass spectroscopy or other proteomics-based approaches.

Low levels of BDNF and TrkB are reported within vulnerable neuronal populations in the brain of AD patients and administration of BDNF or mimetics prevented and reversed symptomatology of AD in animal models^44^,^45^,^46^. Yet, implementation of BDNF as therapy faces numerous methodological and safety issues^47^,^48^. Downstream targets of BDNF and corticosterone signaling like the enzyme DUSP1 could have more targeted therapeutic effects. Design of small molecules selective for DUSP1 is likely difficult given that the catalytic site across different phosphatases is usually very similar^49^. One alternative approach is the titration of DUSP1 levels directly in diseased tissues. Previous overexpression of DUSP1 with viral vectors in the striatum of a mouse model of Huntington’s disease provided neuroprotective effects^50^. In contrast, overexpression of DUSP1 in the hippocampus caused depressive-like phenotypes in mice likely due to suppression of MAPK signaling^51^. In our hands, expression of DUSP1 with a minigene containing self-regulatory elements provides protective effects against dendritic spine loss and *de novo* phosphorylation of tau in the model of chronic corticosterone administration.

A unique feature of the DUSP family of proteins is to dephosphorylate both the tyrosine and threonine residues within a conserved TxY motif, a consensus sequence found in the catalytic core of MAPKs. Phosphorylation of the TxY motif at both residues is required for full MAPK activity^52^. The strength of MAPK signaling frequently determines distinctive outcomes through the duration of its activities. MAPK pathways, including ERK, JNK and p38 pathways, are activated in vulnerable neurons in patients with AD suggesting that MAPK pathways are involved mechanistically in the pathophysiology of AD^53^. For instance, several studies suggest that amyloid deposits, a pathological feature of AD, can trigger activation of JNK and p38 with associated tau pathology that were reduced following immunization trials against amyloid-β peptides in both Tg2576 AD mouse model and human AD brains^54^. While many studies have concentrated on the activation of MAPK signaling, the mechanisms that lead to its termination are less studied. DUSP1 dephosphorylates and inactivates preferably p38 and JNK and, to a lesser extent, ERK1/2^55^ and may be a viable target for AD therapies given the difficulties associated with the amyloid reduction clinical trials to date^56^,^57^.

Broad-spectrum substrates of MAPKs implicated in AD include the cytoskeletal protein tau. We found that DUSP1 overexpression decreased tau phosphorylation whereas DUSP1 knockdown increased tau phosphorylation. Cycles of phosphorylation/ dephosphorylation of tau in dendrites are postulated to be involved in synaptic remodeling, neuronal plasticity, and correlate highly with cognitive decline and synaptic dysfunction during the progression of AD^58^,^59^. Hyperphosphorylated tau proteins are the products of an imbalance between phosphorylation and dephosphorylation, reducing the abilities of tau to bind and stabilize microtubule networks^60^. Instead, hyperphosphorylated tau proteins can self-aggregate in filaments which accumulation positively correlates with cognitive deficits and neurodegeneration^60^. The fact that mice with genetic invalidation of tau survive in adulthood somewhat normally indicates that hyperphosphorylation of tau provides a gain-of-function more deleterious than the loss-of-function. For instance, knockout of tau blocks the behavioral, morphological and biochemical responses to chronic glucocorticoid administration or chronic stress, suggesting that hyperphosphorylated tau may deviate neuroplasticity from adaptation to maladaptation^14^.

Stress and glucocorticoids increase tau phosphorylation in dendrites and synapses commensurate to the intensity of stimuli^13^,^14^. It is hypothesized that stress and glucocorticoid-induced tau phosphorylation is transient or reversible. Expression of *DUSP1* shares features of immediate early genes with low baseline levels, rapid induction and turnover that is responsive to stress and glucocorticoids^61^. We found that deficits of DUSP1 expression introduced signs of tauopathy and synaptopathy in pyramidal neurons of mouse cortex. The evidence point toward DUSP1 chronic deficiency in maladaptation of cortical neurons to insufficient signaling of GR. Contrary to PP2A^60^, DUSP1 is not a direct tau phosphatase. Yet, DUSP1 could permit deactivation of kinases including p38 and JNK that phosphorylate tau^62^.

Strategies aiming at restoring DUSP1 functions could provide mechanistic insights for preventing or lessening AD neuropathology. This requires methods for delivering DUSP1 functions in a time-controlled manner. One approach with great potential would be to stop the aggravation of AD neuropathology upon chronic stress/corticosterone by providing the necessary neurotrophic support that the diseased brain lacks to restore sufficient GR signaling.

## Materials and Methods

A list of the reagents used is provided as Supplementary Table 1.

### Mouse Studies

Time-pregnant CD1 mice (Janvier Laboratories) were allowed *ad libitum* access to food, water, and maintained on a 12-h light-dark cycle (onset of the light cycle 5:00 AM). Solutions of corticosterone (15 mg/kg) and vehicle (40% dimethyl-formamide, 60% sesame oil) were made fresh daily and always injected systemically in the mornings for 10 consecutive days starting on post-natal day (PND) 21 onwards. 1-(1,1-Dimethylethyl)-3-(1-naphthalenyl)-1H-pyrazolo[3,4-*d*]pyrimidin-4-amine (1NaPP1, 25 mM) was administered via the drinking water for 10 consecutive days from P21 onwards to block the ATP binding site of the TrkB mutant F669A^28^. Five μl of blood was collected from the tail vein in the morning at about 7:30 AM, 9:30 AM, at mid day at 12:30 PM and in the evening at 7:30 PM. Blood levels of corticosterone were measured by ELISA following manufacturer’s instructions (Enzo). Cortical levels of BDNF were also measured by ELISA (Promega). Mice were subjected to a tail suspension test (TST) around noon on the day before sacrifice. The tail was taped 5 minutes before suspension to a hook located in a 56-cm × 40-cm × 33-cm dark box. Frontal visual inspection of immobility postures was measured using a timer during a period of 9 min. Immobility was scored as described^63^. Measures were taken to minimize pain and discomfort for the animals with respect to the 3R regulations. All experiments followed European Union (Council directive 86/609EEC) and institutional guidelines for laboratory animal care and use. Institutional license for hosting animals was approved by the French Ministry of Agriculture (No. D34-172-13). The animal experiment protocols were approved by the French ministry for education and scientific research and by the local ethical committee (authorization number CEEA-LR-00651-01).

### *In-utero* electroporation

One μg of DNA was injected in the ventricle at E15-E15.5 on CD1 mouse embryos *in utero* and electroporated (NEPA21, Nepagene, 30 V, pON 50 msec, pOFF 950 msec, 5 pulses) as described^64^. DNA constructs carrying a green fluorescent protein (GFP) reporter cassette were introduced into the neuronal lineage of the cortical plate that specifies future layer LII/III excitatory neurons, and allowed the embryos to develop till one month of age before analysis (Supplementary Figure S1A). Expression of GFP transgene tag is detectable in scattered non-overlapping neurons of the newborns mice for up to two month of age.

### Plasmids and production of lentivirus

The shRNA plasmids against mouse DUSP1, GR, TrkB, scramble control and molecular replacement vectors for the mutant GR and TrkB were previously described^28^. DUSP1 minigene was amplified by PCR from a bank of total cDNA prepared from total RNA extracted from somatosensory cortex and cloned into the TOPO plasmid (Invitrogen). A CMV-GFP expressing cassette was also included in the recombinant vector. Site-directed mutagenesis with Quickchange (Agilent) was used to fused the RFP to DUSP1 C-terminus. All constructs were verified by sequencing. Lentiviruses were produced in 293FT cells by co-transfecting recombinant FCIV1, Δ8.9 and pCMV-VSVG plasmids. Media were collected after 48 h and diluted 1:4 with regular culture medium to infect primary cortical cultures at DIV0.

### Immunohistochemistry

Brain hemisections were fixed with 4% ice-cold paraformaldehyde (PFA) for 2 h and equilibrated in 30% sucrose. Free-floating coronal sections rinsed in phosphate-buffered-saline (PBS) were blocked in 5% normal goat serum, 5% normal horse serum, PBS, 0.1% triton X-100 for 2 h at 25 °C. Primary antibodies (p-S287 1.5 μg/ml; p-S155 1.5 μg/ml; GR 1:100; GFP 1:2,000; RFP 1:1,000, PHF1 1:500) were incubated for 2 days. PHF1 (S396/S404) is a progressive marker of tau pathology, which phosphorylation by stress-activated MAPK responds to glucocorticoids^13^. Alexa fluor-conjugated secondary antibodies (Molecular Probes 1:2,000) were incubated for 2 h at 25 °C. Optical densities were obtained on grayscale confocal images in select regions of interest and further divided by surface area. Data were averaged per brain slices, per mice, and per group.

### Dendritic Spine Studies

Fluorescence images were taken on a LSM510 laser-scanning confocal microscope (Carl-Zeiss) equipped with x63 Plan Neofluor NA1.3 oil-immersion objective and digital zoom 8. Z-stack images were processed using ImageJ (Supplementary Figure S1E). Laser excitation, fluorescence emission capture, pinhole were held constant throughout the study. Dendritic segments included in the analyses met the following criteria: (1) be parallel or at acute angles relative to the coronal surface of sections to allow unambiguous identification of spines, (2) segments had no overlap with other branches, (3) dendritic segments from apical tree were imaged within the first 100 μm from the pial surface, and (4) dendritic segments from the basal tree were captured between approximately 50–150 μm distance from the soma. About 200 dendritic spines from at least 20–30 dendritic segments were counted per condition, and averaged per animal and groups as described^28^.

### Brain tissue lysate processing

Punches of the somatosensory cortex were obtained from 200 μm-thick mouse brain hemisections on ice using a dissecting tool (Stoelting) and frozen in liquid nitrogen. For Western blot analysis, tissue was lysed in 10 mM Tris-HCl, pH = 8.0, 150 mM NaCl, 1 mM EDTA, 10% glycerol, 1% NP40, 0.1% SDS, 0.1% triton X-100 complemented with protease inhibitors, 1 mM Na_3_VO_4_, 10 mM NaF, 10 nM calyculin A and cleared from debris by centrifugation (14,000 rpm for 10 min) as previously described^26^. RNA extraction was performed with Trizol according to manufacturer’s instruction (Invitrogen).

### Quantitative real-time PCR

Hot-start SYBR Green PCR kit (Qiagen) was used in 16 μL reactions containing 1 μL of cDNA, and 1 μL of 5 pmol μL^−1^ primer mixture. qPCR was performed with ABI 7900 instrument (Applied Biosystems), followed by melt-curve analysis. Fold changes in gene expression were calculated using ΔΔCt (Ct = cycle number at threshold) analytical method that includes normalization against housekeeping gene^65^. The primers against mouse genes are described in the Supplementary Table S1.

### Cell culture and lysis

Primary E18 cortical neurons were prepared from time-pregnant CD1 mice, cultured on poly-D-lysine, and maintained for 2 weeks *in vitro* in Neurobasal medium containing B27 supplement, 0.5 mM L-glutamine, 5-fluorouridine and uridine (10 μM each). 293FT cells (Invitrogen) were grown in DMEM containing 10% FBS plus 200 μg/ml G418. Cells lysates were prepared in 10 mM Tris-HCL, pH = 8.0, 150 mM NaCl, 1 mM EDTA, 10% glycerol, 1% NP40, 0.1% SDS plus protease inhibitors, 1 mM Na_3_VO_4_, 10 nM calyculin A and 10 mM NAF and cleared (14,000 rpm for 10 min).

### Human Studies

Frozen tissues of the frontal cortex [Broadman area BA9 and BA10] of 71 cases with age at death ranging from 29 to 98 years, and postmortem interval (PMI) ≤ 36 hours were obtained from the Rush Religious Orders Study (RROS), the University of Pennsylvania Brain Bank (Center for Neurodegenerative Disease Research), the Harvard Brain Bank (Harvard Brain Tissue Resource Center), and the Emory Brain Bank (Center for Neurodegenerative Disease). The cases were clinically examined and diagnosed as normal controls with no cognitive impairment, mild cognitive impairment (MCI) insufficient to meet criteria for dementia, and AD. Cognitive assessment scores were collected within one year prior to death using the Mini-Mental State Exam (MMSE)^66^. Exclusion criteria included argyrophilic grain disease, frontotemporal dementia, Lewy body disease, mixed dementias, Parkinson’s disease, and stroke. A board certified neuropathologist blinded to the clinical diagnosis performed a neuropathological diagnosis based on established criteria^67^. Tissue samples were processed as described^68^ and Western blots repeated 5 times for each sample to minimize experimental bias. Digitalized data were measured by optical densitometric analyses with NIH imageJ, subtracted of background and normalized to GAPDH levels. Analyses were not corrected for drug treatment. Demographic information is provided in Supplementary Figure S3. The study is performed under the auspices of guidelines administrated by the Nathan Kline Institute and the New York University Langone Medical Center.

### Statistical Analysis

Data were compared using Student’s t-test or correlated with Pearson r for comparing 2 variables. For multiple comparisons, we used 2-way ANOVA post-hoc Tukey’s test (GraphPad Prism). Statistic tables are presented in the supplementary information.

## Additional Information

**How to cite this article**: Arango-Lievano, M. *et al.* Deletion of Neurotrophin Signaling through the Glucocorticoid Receptor Pathway Causes Tau Neuropathology. *Sci. Rep.* **6**, 37231; doi: 10.1038/srep37231 (2016).

**Publisher’s note:** Springer Nature remains neutral with regard to jurisdictional claims in published maps and institutional affiliations.

## Supplementary Material

Supplementary Information

## References

[b1] Oitzl M. S., Champagne D. L., van der Veen R. & de Kloet E. R. Brain development under stress: hypotheses of glucocorticoid actions revisited. Neurosci Biobehav Rev 34, 853–866, doi: S0149-7634(09)00103-1/j.neubiorev.2009.07.006 (2010). 19631685 10.1016/j.neubiorev.2009.07.006

[b2] Pariante C. M. Risk factors for development of depression and psychosis. Glucocorticoid receptors and pituitary implications for treatment with antidepressant and glucocorticoids. Ann N Y Acad Sci 1179, 144–152, doi: NYAS4978/j.1749-6632.2009.04978.x (2009). 19906237 10.1111/j.1749-6632.2009.04978.x PMC2982725

[b3] Fardet L., Petersen I. & Nazareth I. Suicidal behavior and severe neuropsychiatric disorders following glucocorticoid therapy in primary care. Am J Psychiatry 169, 491–497, doi: 10.1176/appi.ajp.2011.11071009 (2012). 22764363

[b4] Lucassen P. J. *et al.* Neuropathology of stress. Acta neuropathologic0 127, 109–135, doi: 10.1007/s00401-013-1223-5 (2014). PMC3889685 24318124

[b5] Karatsoreos I. N. & McEwen B. S. Psychobiological allostasis: resistance, resilience and vulnerability. Trends Cogn Sci 15, 576–584, doi: 10.1016/j.tics.2011.10.005 (2011). 22078931

[b6] Chattarji S., Tomar A., Suvrathan A., Ghosh S. & Rahman M. M. Neighborhood matters: divergent patterns of stress-induced plasticity across the brain. Nat Neurosci 18, 1364–1375, doi: 10.1038/nn.4115 (2015). 26404711

[b7] Gourley S. L. *et al.* Action control is mediated by prefrontal BDNF and glucocorticoid receptor binding. Proc Natl Acad Sci USA 109, 20714–20719, doi: 10.1073/pnas.1208342109 (2012). 23185000 PMC3528547

[b8] Dias-Ferreira E. *et al.* Chronic stress causes frontostriatal reorganization and affects decision-making. Science 325, 621–625, doi: 325/5940/621/science.1171203 (2009). 19644122 10.1126/science.1171203

[b9] Sapolsky R. M. Stress and the brain: individual variability and the inverted-U. Nat Neurosci 18, 1344–1346, doi: 10.1038/nn.4109 (2015). 26404708

[b10] Jeanneteau F. & Arango-Lievano M. Linking Mitochondria to Synapses: New Insights for Stress-Related Neuropsychiatric Disorders. Neural Plast 2016, 3985063, doi: 10.1155/2016/3985063 (2016). 26885402 PMC4738951

[b11] Jeanneteau F. & Chao M. V. Are BDNF and glucocorticoid activities calibrated? Neuroscience 239, 173–195 (2013). 23022538 10.1016/j.neuroscience.2012.09.017 PMC3581703

[b12] Liston C. *et al.* Circadian glucocorticoid oscillations promote learning-dependent synapse formation and maintenance. Nature Neuroscience 16, 698–705 (2013). 23624512 10.1038/nn.3387 PMC3896394

[b13] Sotiropoulos I. *et al.* Stress acts cumulatively to precipitate Alzheimer’s disease-like tau pathology and cognitive deficits. J Neurosci 31, 7840–7847, doi: 10.1523/JNEUROSCI.0730-11.2011 (2011). 21613497 PMC6633145

[b14] Lopes S. *et al.* Tau protein is essential for stress-induced brain pathology. Proc Natl Acad Sci USA, doi: 10.1073/pnas.1600953113 (2016). PMC4932951 27274066

[b15] Papadopoulou A. *et al.* Acute and chronic stress differentially regulate cyclin-dependent kinase 5 in mouse brain: implications to glucocorticoid actions and major depression. Translational psychiatry 5, e578, doi: 10.1038/tp.2015.72 (2015). 26057048 PMC4490283

[b16] Biddie S. C., Conway-Campbell B. L. & Lightman S. L. Dynamic regulation of glucocorticoid signalling in health and disease. Rheumatology 51, 403–412, doi: 10.1093/rheumatology/ker215 (2012). 21891790 PMC3281495

[b17] Cohen S. *et al.* Chronic stress, glucocorticoid receptor resistance, inflammation, and disease risk. Proceedings of the National Academy of Sciences of the United States of America 109, 5995–5999, doi: 10.1073/pnas.1118355109 (2012). 22474371 PMC3341031

[b18] Hodes G. E., Kana V., Menard C., Merad M. & Russo S. J. Neuroimmune mechanisms of depression. Nat Neurosci 18, 1386–1393, doi: 10.1038/nn.4113 (2015). 26404713 PMC4843114

[b19] Barnes P. J. & Adcock I. M. Glucocorticoid resistance in inflammatory diseases. Lancet 373, 1905–1917, doi: 10.1016/S0140-6736(09)60326-3 (2009). 19482216

[b20] Menke A. *et al.* Dexamethasone stimulated gene expression in peripheral blood is a sensitive marker for glucocorticoid receptor resistance in depressed patients. Neuropsychopharmacology: official publication of the American College of Neuropsychopharmacology 37, 1455–1464, doi: 10.1038/npp.2011.331 (2012). 22237309 PMC3327850

[b21] Holsboer F. & Ising M. Stress hormone regulation: biological role and translation into therapy. Annu Rev Psychol 61, 81–109, C101-111, doi: 10.1146/annurev.psych.093008.100321 (2010). 19575614

[b22] McEwen B. S. *et al.* Mechanisms of stress in the brain. Nat Neurosci 18, 1353–1363, doi: 10.1038/nn.4086 (2015). 26404710 PMC4933289

[b23] Arango-Lievano M. & Jeanneteau F. Timing and crosstalk of glucocorticoid signaling with cytokines, neurotransmitters and growth factors. Pharmacological research 113, 1–17, doi: 10.1016/j.phrs.2016.08.005 (2016). 27498156

[b24] Chen D. Y., Bambah-Mukku D., Pollonini G. & Alberini C. M. Glucocorticoid receptors recruit the CaMKIIalpha-BDNF-CREB pathways to mediate memory consolidation. Nat Neurosci 15, 1707–1714, doi: nn.3266/nn.3266 (2012). 23160045 10.1038/nn.3266 PMC3509234

[b25] Revest J. M. *et al.* BDNF-TrkB signaling through Erk1/2 phosphorylation mediates the enhancement of fear memory induced by glucocorticoids. Mol Psychiatry, doi: 10.1038/mp.2013.134 (2013). PMC4195976 24126929

[b26] Jeanneteau F. *et al.* BDNF and glucocorticoids control corticotrophin-releasing hormone (CRH) homeostasis in the hypothalamus. PNAS 109, 1305–1310 (2012). 22232675 10.1073/pnas.1114122109 PMC3268297

[b27] Lambert W. M. *et al.* BDNF-signaling rewrites the glucocorticoid transcriptome via glucocorticoid receptor phosphorylation. Molecular and Cellular Biology 33, 3700–3714 (2013). 23878391 10.1128/MCB.00150-13 PMC3753865

[b28] Arango-Lievano M. *et al.* Neurotrophic-priming of glucocorticoid receptor signaling is essential for neuronal plasticity to stress and antidepressant treatment. Proc Natl Acad Sci USA 112, 15737–15742, doi: 10.1073/pnas.1509045112 (2015). 26630005 PMC4697403

[b29] Bath K. G., Schilit A. & Lee F. S. Stress effects on BDNF expression: effects of age, sex, and form of stress. Neuroscience 239, 149–156, doi: 10.1016/j.neuroscience.2013.01.074 (2013). 23402850

[b30] Gray J. D., Milner T. A. & McEwen B. S. Dynamic plasticity: The role of glucocorticoids, brain-derived neurotrophic factor and other trophic factors. Neuroscience 239, 214–227, doi: 10.1016/j.neuroscience.2012.08.034 (2013). 22922121 PMC3743657

[b31] Jeanneteau F., Deinhardt K., Miyoshi G., Bennett A. M. & Chao M. V. The MAP kinase phosphatase MKP-1 regulates BDNF-induced axon branching. Nat Neurosci 13, 1373–1379, doi: nn.2655/nn.2655 (2010). 20935641 10.1038/nn.2655 PMC2971689

[b32] Doi M. *et al.* Light-inducible and clock-controlled expression of MAP kinase phosphatase 1 in mouse central pacemaker neurons. J Biol Rhythms 22, 127–139, doi: 22/2/127/0748730406298332 (2007). 17440214 10.1177/0748730406298332

[b33] de Quervain D. J. *et al.* Glucocorticoid-related genetic susceptibility for Alzheimer’s disease. Hum Mol Genet 13, 47–52, doi: 10.1093/hmg/ddg361ddg361 (2004). 14583441

[b34] Green K. N., Billings L. M., Roozendaal B., McGaugh J. L. & LaFerla F. M. Glucocorticoids increase amyloid-beta and tau pathology in a mouse model of Alzheimer’s disease. J Neurosci 26, 9047–9056, doi: 26/35/9047/JNEUROSCI.2797-06.2006 (2006). 16943563 10.1523/JNEUROSCI.2797-06.2006 PMC6675335

[b35] Menke A. *et al.* Genetic variation in FKBP5 associated with the extent of stress hormone dysregulation in major depression. Genes, brain, and behavior 12, 289–296, doi: 10.1111/gbb.12026 (2013). 23406438

[b36] Proctor D. T., Coulson E. J. & Dodd P. R. Reduction in post-synaptic scaffolding PSD-95 and SAP-102 protein levels in the Alzheimer inferior temporal cortex is correlated with disease pathology. Journal of Alzheimer’s disease: JAD 21, 795–811, doi: 10.3233/JAD-2010-100090 (2010). 20634587

[b37] Alldred M. J., Duff K. E. & Ginsberg S. D. Microarray analysis of CA1 pyramidal neurons in a mouse model of tauopathy reveals progressive synaptic dysfunction. Neurobiol Dis 45, 751–762, doi: 10.1016/j.nbd.2011.10.022 (2012). 22079237 PMC3259262

[b38] Elgh E. *et al.* Cognitive dysfunction, hippocampal atrophy and glucocorticoid feedback in Alzheimer’s disease. Biol Psychiatry 59, 155–161, doi: 10.1016/j.biopsych.2005.06.017 (2006). 16125145

[b39] Csernansky J. G. *et al.* Plasma cortisol and progression of dementia in subjects with Alzheimer-type dementia. Am J Psychiatry 163, 2164–2169, doi: 10.1176/ajp.2006.163.12.2164 (2006). 17151169 PMC1780275

[b40] Swaab D. F., Bao A. M. & Lucassen P. J. The stress system in the human brain in depression and neurodegeneration. Ageing research reviews 4, 141–194, doi: 10.1016/j.arr.2005.03.003 (2005). 15996533

[b41] Notarianni E. Hypercortisolemia and glucocorticoid receptor-signaling insufficiency in Alzheimer’s disease initiation and development. Curr Alzheimer Res 10, 714–731 (2013). 23906001 10.2174/15672050113109990137

[b42] Brureau A. *et al.* Deregulation of hypothalamic-pituitary-adrenal axis functions in an Alzheimer’s disease rat model. Neurobiology of aging 34, 1426–1439, doi: 10.1016/j.neurobiolaging.2012.11.015 (2013). 23273603

[b43] Adzic M. *et al.* Acute or chronic stress induce cell compartment-specific phosphorylation of glucocorticoid receptor and alter its transcriptional activity in Wistar rat brain. J Endocrinol 202, 87–97, doi: 10.1677/JOE-08-0509 (2009). 19406955 PMC2695659

[b44] Nagahara A. H. *et al.* Neuroprotective effects of brain-derived neurotrophic factor in rodent and primate models of Alzheimer’s disease. Nat Med 15, 331–337, doi: nm.1912/nm.1912 (2009). 19198615 10.1038/nm.1912 PMC2838375

[b45] Devi L. & Ohno M. 7,8-dihydroxyflavone, a small-molecule TrkB agonist, reverses memory deficits and BACE1 elevation in a mouse model of Alzheimer’s disease. Neuropsychopharmacology 37, 434–444, doi: 10.1038/npp.2011.191 (2012). 21900882 PMC3242305

[b46] Devi L. & Ohno M. TrkB reduction exacerbates Alzheimer’s disease-like signaling aberrations and memory deficits without affecting beta-amyloidosis in 5XFAD mice. Translational psychiatry 5, e562, doi: 10.1038/tp.2015.55 (2015). 25942043 PMC4471286

[b47] Nagahara A. H. & Tuszynski M. H. Potential therapeutic uses of BDNF in neurological and psychiatric disorders. Nat Rev Drug Discov 10, 209–219, doi: 10.1038/nrd3366 (2011). 21358740

[b48] Song J. H., Yu J. T. & Tan L. Brain-Derived Neurotrophic Factor in Alzheimer’s Disease: Risk, Mechanisms, and Therapy. Mol Neurobiol 52, 1477–1493, doi: 10.1007/s12035-014-8958-4 (2015). 25354497

[b49] Jeffrey K. L., Camps M., Rommel C. & Mackay C. R. Targeting dual-specificity phosphatases: manipulating MAP kinase signalling and immune responses. Nat Rev Drug Discov 6, 391–403, doi: nrd2289/nrd2289 (2007). 17473844 10.1038/nrd2289

[b50] Taylor D. M. *et al.* MAP kinase phosphatase 1 (MKP-1/DUSP1) is neuroprotective in Huntington’s disease via additive effects of JNK and p38 inhibition. J Neurosci 33, 2313–2325, doi: 10.1523/JNEUROSCI.4965-11.2013 (2013). 23392662 PMC3711389

[b51] Duric V. *et al.* A negative regulator of MAP kinase causes depressive behavior. Nat Med 16, 1328–1332, doi: nm.2219/nm.2219 (2010). 20953200 10.1038/nm.2219 PMC3066515

[b52] Boutros T., Chevet E. & Metrakos P. Mitogen-activated protein (MAP) kinase/MAP kinase phosphatase regulation: roles in cell growth, death, and cancer. Pharmacol Rev 60, 261–310, doi: 60/3/261/pr.107.00106 (2008). 18922965 10.1124/pr.107.00106

[b53] Zhu X., Lee H. G., Raina A. K., Perry G. & Smith M. A. The role of mitogen-activated protein kinase pathways in Alzheimer’s disease. Neuro-Signals 11, 270–281, doi: 67426 (2002). 12566928 10.1159/000067426

[b54] Ferrer I. Stress kinases involved in tau phosphorylation in Alzheimer’s disease, tauopathies and APP transgenic mice. Neurotoxicity research 6, 469–475 (2004). 15658002 10.1007/BF03033283

[b55] Owens D. M. & Keyse S. M. Differential regulation of MAP kinase signalling by dual-specificity protein phosphatases. Oncogene 26, 3203–3213, doi: 1210412/sj.onc.1210412 (2007). 17496916 10.1038/sj.onc.1210412

[b56] Panza F., Logroscino G., Imbimbo B. P. & Solfrizzi V. Is there still any hope for amyloid-based immunotherapy for Alzheimer’s disease? Current opinion in psychiatry 27, 128–137, doi: 10.1097/YCO.0000000000000041 (2014). 24445401

[b57] Soejitno A., Tjan A. & Purwata T. E. Alzheimer’s Disease: Lessons Learned from Amyloidocentric Clinical Trials. CNS drugs 29, 487–502, doi: 10.1007/s40263-015-0257-8 (2015). 26187557

[b58] Rissman R. A. Stress-induced tau phosphorylation: functional neuroplasticity or neuronal vulnerability? Journal of Alzheimer’s disease: JAD 18, 453–457, doi: 10.3233/JAD-2009-1153 (2009). 19584431 PMC2906152

[b59] Mufson E. J. *et al.* Molecular and cellular pathophysiology of preclinical Alzheimer’s disease. Behav Brain Res 311, 54–69, doi: 10.1016/j.bbr.2016.05.030 (2016). 27185734 PMC4931948

[b60] Iqbal K., Liu F. & Gong C. X. Tau and neurodegenerative disease: the story so far. Nat Rev Neurol 12, 15–27, doi: 10.1038/nrneurol.2015.225 (2016). 26635213

[b61] Jeanneteau F. & Deinhardt K. Fine-tuning MAPK signaling in the brain: The role of MKP-1. Communicative and Integrative Biology 4, 1–3 (2011). 21980558 10.4161/cib.4.3.14766 PMC3187886

[b62] Correa S. A. & Eales K. L. The Role of p38 MAPK and Its Substrates in Neuronal Plasticity and Neurodegenerative Disease. Journal of signal transduction 2012, 649079, doi: 10.1155/2012/649079 (2012). 22792454 PMC3389708

[b63] Can A. *et al.* The tail suspension test. J Vis Exp e3769, doi: 3769/3769 (2012). 22315011 10.3791/3769 PMC3353516

[b64] Langevin L. M. *et al.* Validating in utero electroporation for the rapid analysis of gene regulatory elements in the murine telencephalon. Dev Dyn 236, 1273–1286, doi: 10.1002/dvdy.21126 (2007). 17377980

[b65] Arango-Lievano M. *et al.* Cell-Type Specific Expression of p11 Controls Cocaine Reward. Biol Psychiatry, doi: 10.1016/j.biopsych.2014.02.012 (2014). PMC4145045 24725970

[b66] Folstein M. F., Folstein S. E. & McHugh P. R. “Mini-mental state”. A practical method for grading the cognitive state of patients for the clinician. J Psychiatr Res 12, 189–198 (1975). 1202204 10.1016/0022-3956(75)90026-6

[b67] Braak H. & Braak E. Neuropathological stageing of Alzheimer-related changes. Acta neuropathologica 82, 239–259 (1991). 1759558 10.1007/BF00308809

[b68] Counts S. E. *et al.* Reduction of cortical TrkA but not p75(NTR) protein in early-stage Alzheimer’s disease. Annals of neurology 56, 520–531, doi: 10.1002/ana.20233 (2004). 15455399

