# Supplementary material for: Deletion of Neurotrophin Signaling through the Glucocorticoid Receptor Pathway Causes Tau Neuropathology
Source: Sci Rep. 2016 Nov 16;6:37231. doi: 10.1038/srep37231 (PMC5110980; doi:10.1038/srep37231)
Supplement: Supplementary Information [file srep37231-s1.pdf]

Deletion of Neurotrophin Signaling through the Glucocorticoid Receptor Pathway Causes Tau Neuropathology

Margarita Arango-Lievano[1,2,3*], Camille Peguet[1,2,3], Matthias Catteau[1,2,3], Marie-Laure Parmentier[1,2,3], Synphen Wu[4], Moses V Chao[4], Stephen D. Ginsberg[5], Freddy Jeanneteau[1,2,3*]

[1]Inserm, U1191, Institute of Functional Genomics, F-34000 Montpellier, France, [2]CNRS, UMR-5203, F-34000 Montpellier, France, [3]Université de Montpellier, F-34000 Montpellier, France, [4]Skirball Institute of biomolecular medicine, New York University Langone Medical Center, New York, NY 10016, USA, [5]Center for Dementia Research, Nathan Kline Institute, Departments of Psychiatry, Neuroscience & Physiology, New York University Langone Medical Center, Orangeburg, NY 10962, USA

SUPPLEMENTARY INFORMATION

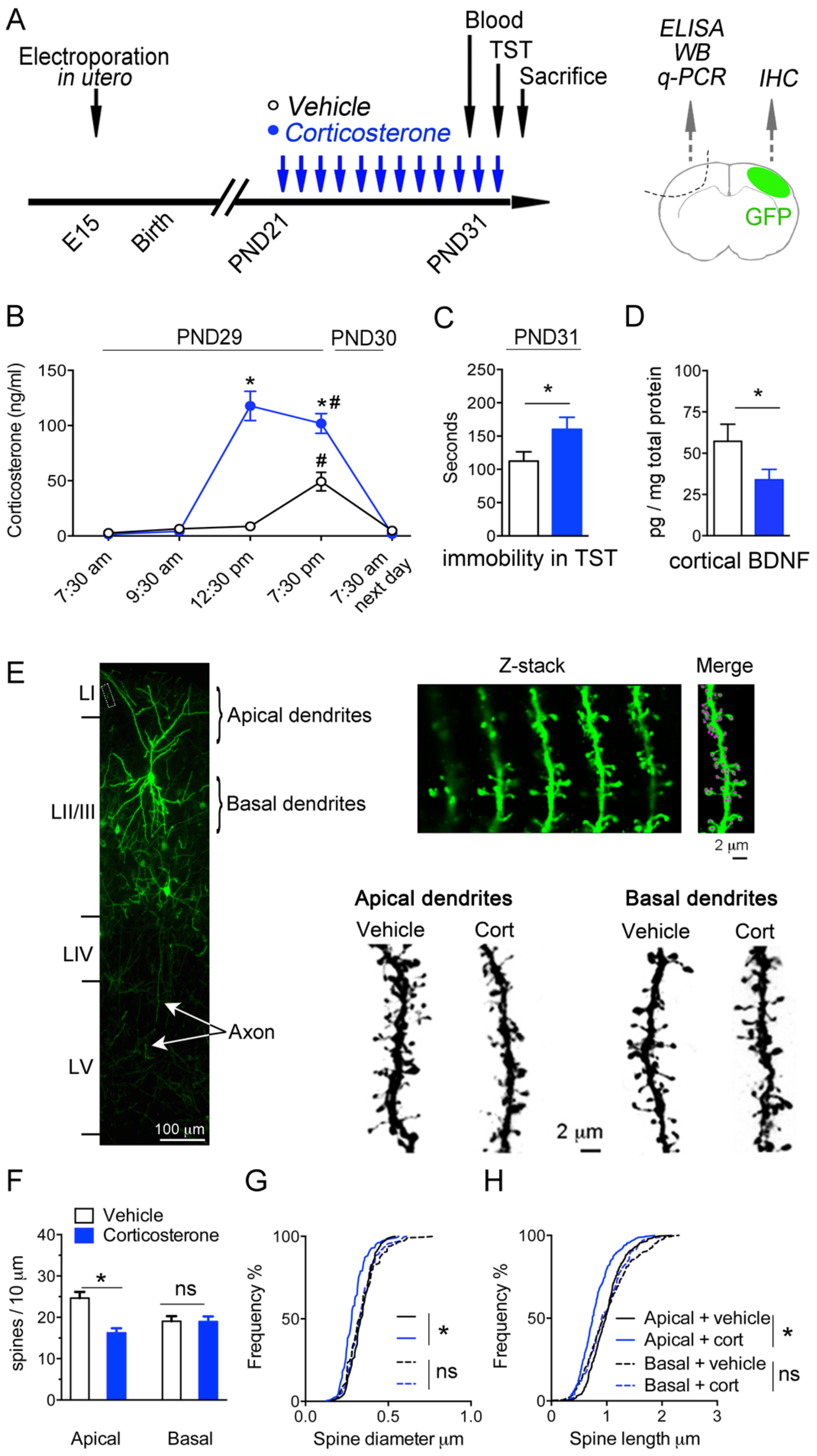

Supplementary Figure S1. Mouse model of glucocorticoid resistance. (A) Experimental timeline. After perfusion with PBS, the electroporated hemisphere was fixed in PFA and

somatosensory cortex of contralateral hemisphere dissected immediately and frozen. (B) Levels of corticosterone in whole blood collected before (7:30 AM, 9:30 AM) and after (12:30 PM, 7:30 PM and 7:30 AM on the next day) the ninth daily injection of 15mg/kg synthetic corticosterone. Mice were handled and habituated daily, starting one week before blood collection to limit the impact of novelty and stress on corticosterone blood levels. Mean ± SEM of at least N = 5 mice / group; unpaired t-test: vehicle group versus CORT group *P=0.0003, 7:30 AM versus 7:30 PM #P<0.0001. (C) Mice treated with chronic corticosterone spent more time immobile in the tail suspension test compared to mice treated with a vehicle control. Mean ± SEM of N=13 controls and 12 corticosterone mice, unpaired t-test P<0.05 (*). (D) Cortical amounts of BDNF protein (Mean ± SEM) measured by ELISA and normalized to total amount of protein (unpaired t-test * P=0.035, N = 10 mice/ group). (E) Layer II/III excitatory neuron of somatosensory cortex expressing GFP are polarized, sending apical tufts in the layer I, basal dendritic territories in the layer II/III and axon in deeper layers. Confocal Z-stack of dendrites allows analyses of spine number and size. (F) Spine density (Mean ± SEM) at apical and basal dendrites. Unpaired t-test * P=0.0018, N=5 mice/group. Distribution of dendritic spines according to their diameter (G) and length (H). Kolmogorov-Smirnov test * P<0.0001, N>200 spines/ group of 5 mice each.

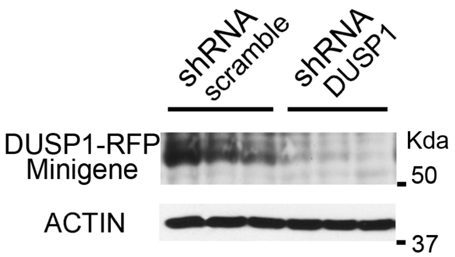

Supplementary Figure S2. Knockdown of DUSP1. Validation of the shRNA against DUSP1 in HEK293 cells transfected with the recombinant DUSP1-RFP minigene. Western blot represents RFP immunoreactivity. Each lane represents an independent sample.

A

Cohort	Diagnosis	Age	Gender	PMI (h)	BRAIN Weight (g)	Braak stage	MMSE score	Mean ± sem (DUSP1)
1	CTR, Healthy	72.2 ± 4.89	6M/ 4F	13.85 ± 2.4	1273 ± 50	1.58 ± 0.3	n.d.	4.693 ± 0.54
	AD	77.8 ± 2.68	8M/ 7F	9.13 ± 1.24	1116 ± 44 *	5.7 ± 0.12 ***	n.d.	8.488 ± 1.688 *
2	CTR, Healthy	72.5 ± 3.9	11M/ 6F	12.3 ± 1.8	1315 ± 43	1.29 ± 0.3	28.8 ± 0.41	0.596 ± 0.05
	AD, Mild impairment	74.8 ± 9.5	1M/ 4F	15.6 ± 2.3	1105 ± 74 *	5.2 ± 0.2 ***	23.8 ± 0.2 ***	0.476 ± 0.04
	AD, Severe impairment	77.4 ± 1.8	9M/ 15F	11.9 ± 1.2	1127 ± 33 **	5.75 ± 0.09 ***	9.2 ± 1.24 ***	0.414 ± 0.04 **

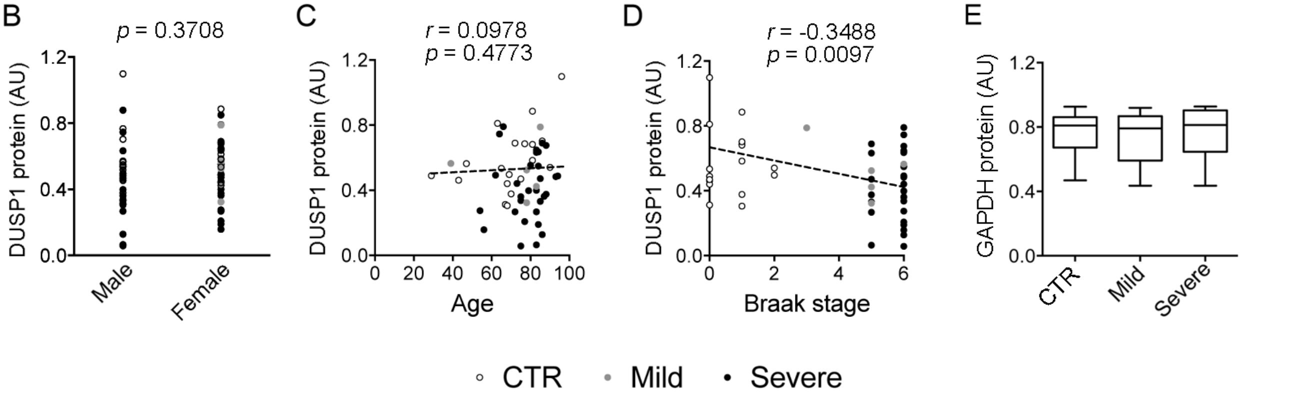

Supplementary Figure S3. Human demographic data. (A) Patients diagnosed with AD (N = 44) and non-demented healthy controls CTR (N = 27) are sorted in 2 cohorts based on cognitive assessment before death with the mini mental state exam (MMSE). MMSE scores are used to sort patients in cohort 2 according to cognitive performance: 5 subjects showed mild cognitive impairment (score between 24 to 19), 24 subjects showed severe cognitive deficits (score between 18 to 0) and 17 subjects showed scores in normal range of healthy individuals (between 30 and 25). Possible confounds are gender (M = male, F = female), age, medication and postmortem interval (PMI). Unpaired t-test comparisons indicated no significant differences of age and PMI between groups. Unpaired t-test comparisons indicated significant effects of diagnosis on brain weight, Braak stage and MMSE score between disease and CTR groups. *P< 0.05, **P<0.01, ***P<0.001. Unfortunately, medication history is unknown. Analyses are not corrected for drug treatment, which can be considered as a possible confound, a common issue with neuropathological studies.

DUSP1 levels are normalized to that of GAPDH for each sample. n.d = not determined. (B) DUSP1 expression normalized to GAPDH as a function of gender. Unpaired t-Test indicates no significant effect of gender on DUSP1 expression. P>0.05 (C) DUSP1 expression normalized to GAPDH as a function of age. Pearson correlation indicates no significant effect of age on DUSP1 expression in a cohort ranging from 29 to 98 years of age. P>0.05 (D) DUSP1 expression normalized to GAPDH as a function of Braak stage. Pearson correlation indicates that DUSP1 and Braak stage co-vary. (E) Levels of housekeeping protein GAPDH is even in all groups. Therefore, GAPDH is proportionate to total amount of proteins loaded in gels. Unpaired t-test P>0.05.

Supplementary table 1: List of reagents.

Antibodies

**Immunogen**	**Details**	**Source**	**Comments**	**Manufacturer**
p-GR	S155-P	Rabbit polyclonal Affinity purified	Use at 1.5 μg/ml	Homemade (Arango-Lievano et al PNAS 2015)
p-GR	S287-P	Rabbit polyclonal Affinity purified	Use at 1.5 μg/ml	Homemade (Arango-Lievano et al PNAS 2015)
GR	M20	Rabbit polyclonal	Use at 1:400	Santa Cruz Biotechnologies
DUSP1	N19	Rabbit polyclonal	Detects human, mouse and rat	Santa Cruz Biotechnologies
EGR1		Rabbit polyclonal		Cell Signaling
GFP		Chicken polyclonal	Use at 1:2000	Abcam
RFP		Rabbit polyclonal	Use at 1:1000	Rockland
TrkB		Mouse monoclonal		Millipore
TAU1		Mouse monoclonal		BD Bioscience
p-TAU[PHF-1]	S396-P/S404-P Phosphorylation by stress-activated MAPKs Marker of later stage tau pathology	Mouse monoclonal	1 :500 Accumulates during disease progression (de Calignon et al. 2012).	Peter Davies (D'Abramo et al Plos One 2013; Petry et al. Plos One 2014)
GAPDH		Mouse monoclonal		Biodesign
SGK1	ab59337	Rabbit monoclonal		Abcam
P11		Goat polyclonal	Use at 1:200	R&D Systems
NR4A1	E6	Mouse monoclonal	Detects rodent NR4A1	Santa Cruz Biotechnologies
FKBP51		Mouse monoclonal		BD Bioscience
PSD95		Mouse monoclonal	clone K28/43	NeuroMab
Actin	A4700	Mouse monoclonal		Sigma

Drugs

**Compound name**	**Effect**	**Working concentration**	**comments**	**Manufacturer**
Corticosterone	glucocorticoid	15 mg/kg in 40% DMF, 60% sesame oil	IP injections in the mornings	Sigma
BDNF	Neurotrophin	50 ng/ml	TrkB ligand	Preprotech
Dexamethasone	Synthetic glucocorticoid	1 μM in vitro	GR agonist	Sigma
1 NaPP1	Competes with the binding of ATP in the catalytic tyrosine kinase domain of the TrkB mutant (F669A in rat or F616A in mouse numbering schemes).	10 ng/ml in vitro 25 mM in drinking water	Derived from PP1. Refreshed every 3 days (Chen et al. Neuron 2005).	Sigma
Uridine		10 mM	Use with 5FU	Sigma
5-fluoro-uridine		10 mM	Block cell proliferation	Sigma
B27	Serum free	2% (v/v)	Culture supplement	Invitrogen

qPCR primers

Gene	Forward primer	Reverse primer
GR	5'-aacttgctttctcctttggcggga-3'	5'-ccaggggtgcaaagctcaatgaaa-3'
FKBP5	5'-gctggcaaacaacacgagag -3'	5'-gaggagggccgagttcatt -3'
EGR1	5'-AGCCCTTCCAGTGTCGAATCTG -3'	5'-GGTATGCCTCTTGCGTTCATCA -3
DUSP1	5'-CAAGGATGCTGGAGGGAGAGT -3'	5'-TGAGGTAAGCAAGGCAGATGGT -3'
GAPDH	5'-cctgcaccaccaactgcttag-3'	5'-ctgtggtcatgagcccttcc-3'
BDNF IV	5'-CAGAGCAGCTGCCTTGATGTT-3'	5'-GCCTTGTCCGTGGACGTTTA-3'
ACTIN	5'-ATGCTCCCCGGGCTGTAT-3'	5'-TCACCCACATAGGAGTCCTTCTG-3'
NR4A1	5'-TGGCTTTGGTGATTGGATTGA-3'	5'-GGAGCCCGTGTCGATCAGT-3
P11	5'-gcgacaaagaccacttgaca-3'	5'-cactggtccaggtccttcat-3'
c-FOS	5'-CAGCCAAGTGCCGGAATC-3'	5'-CAACGCAGACTTCTCATCTTCAAG-3
SGK1	5'- TGGAAAGGTTCTTCTGGCTAGG-3'	5'- CACCAGGAAAGGGTGCTTCA -3'

Supplementary statistic tables

Refers to Figure 1B : Effect of BDNF on the expression of Dex-regulated genes. SEM = standard error of the mean. N = number of independent experiments. Unpaired t-test showed significant difference between the Dex and Dex+BDNF groups. Values in bold face type are significant at *P* < 0.05.

GROUPS	N	Fold change relative to untreated	SEM	COMPARISONS	Unpaired t-test P value	t value
FKBP5:						
Dex	5	4.776	0.375			
Dex+BDNF	5	5.764	0.2682	Dex vs Dex+BDNF	0.0579	2.212
SGK1:						
Dex	3	1.66	0.2955			
Dex+BDNF	3	2.148	0.741	Dex vs Dex+BDNF	0.5741	0.6113
**EGR1:**						
Dex	4	2.557	1.481			
Dex+BDNF	4	15.84	4.139	Dex vs Dex+BDNF	**0.0233**	**3.022**
**NR4A1:**						
Dex	4	3.725	0.4847			
Dex+BDNF	4	14.11	2.668	Dex vs Dex+BDNF	**0.0087**	**3.828**
**P11:**						
Dex	3	1.828	0.0639			
Dex+BDNF	3	3.711	0.1402	Dex vs Dex+BDNF	**0.0003**	**12.22**
**DUSP1:**						
Dex	6	1.254	0.1058			
Dex+BDNF	6	3.629	0.49	Dex vs Dex+BDNF	**0.0008**	**4.731**
FOS:						
Dex	4	1.835	0.2			
Dex+BDNF	4	5.758	1.875	Dex vs Dex+BDNF	0.0828	2.079

Refers to Figure 1D : GR phosphorylation in the mouse cortex. N = number of mice analyzed per group. Unpaired t-test showed significant difference between vehicle- and corticosterone-treated mice for the number of cells positive for GR phosphorylation at S287 and S155. Values in bold face type are significant at *P* < 0.05.

GROUPS	N mice	GR phosphorylation	SEM	Unpaired t-test P value	t value
**GR phosphorylation positive cells/mm² :**					
S287 vehicle-treated	11	314.1	17.54		
S287 corticosterone-treated	11	142.1	13.72	**<0.0001**	**7.722**
S155 vehicle-treated	11	450.6	22.38		
S155 corticosterone-treated	11	74.96	14.41	**< 0.0001**	**14.11**

Refers to Figure 1E : PHF1 phosphorylation in the mouse cortex. N = number of mice analyzed per group. Unpaired t-test showed significant difference between vehicle- and corticosterone-treated mice for somatic intensity of PHF1 phosphorylation. Values in bold face type are significant at *P* < 0.05.

GROUPS	N mice	Intensity / soma	SEM	Unpaired t-test P value	t value
**PHF1 phosphorylation:**					
vehicle-treated	11	2.187	0.3045		
corticosterone-treated	11	8.644	0.8538	**<0.0001**	**7.123**

Refers to Figure 1F : Effect of chronic corticosterone on the expression of GR-regulated genes. SEM = standard error of the mean. N = number of mice. Unpaired t-test showed significant difference between the vehicle and CORT groups. Values in bold face type are significant at *P* < 0.05.

GROUPS	N mice	% expression relative to vehicle	SEM	COMPARISONS	Unpaired t-test P value	t value
**FKBP5:**						
Vehicle	6	100	18.39			
Corticosterone	6	2099	760.1	vehicle vs corticosterone	**0.0252**	**2.629**
SGK1:						
Vehicle	3	100	20			
Corticosterone	3	132	23	vehicle vs corticosterone	0.353	1.05
EGR1:						
Vehicle	6	100	26.77			
Corticosterone	6	153.4	69.34	vehicle vs corticosterone	0.4892	0.718
**NR4A1:**						
Vehicle	7	100	17.25			
Corticosterone	6	16.96	6.76	vehicle vs corticosterone	**0.0015**	**4.204**
P11:						
Vehicle	7	100	12			
Corticosterone	6	91	11.5	vehicle vs corticosterone	0.6026	0.5361
**DUSP1:**						
Vehicle	7	100	10.17			
Corticosterone	5	56.4	12.28	vehicle vs corticosterone	**0.0072**	**2.955**
FOS:						
Vehicle	7	100	16.3			
Corticosterone	6	96.92	72.77	vehicle vs corticosterone	0.9653	0.0445
**BDNF IV:**						
Vehicle	7	100	15.71			
Corticosterone	6	57.85	7.677	vehicle vs corticosterone	**0.0435**	**2.281**
GR:						
Vehicle	6	100	20.43			
Corticosterone	6	101.2	78.81	vehicle vs corticosterone	0.989	0.0141
ACTIN:						
Vehicle	4	100	12.76			
Corticosterone	4	114	5.970	vehicle vs corticosterone	0.3681	0.9385

Refers to Figure 2: Interaction of CORT with GR phosphorylation. Density = number of spines scored on defined dendritic segments. SEM = standard error of the mean. N = number of mice per group. Two-way ANOVA, post-hoc Tukey's test showed that the GR-2A mutant interfered with the effects of CORT when compared to the GR-WT groups. Values in bold face type are significant at *P* < 0.05.

2B Apical spine density:

Two-way ANOVA Table	SS	DF	MS	F (DFn, DFd)	
Effect of GR-2A	43.9	1	43.9	F (1, 20) = 8.752	
Effect of CORT	191.2	1	191.2	F (1, 20) = 38.12	
**GROUPS**	**N mice**	**Density**	**SEM**	**COMPARISONS**	**P value**
GR-WT+vehicle	6	25.35	0.5853	**GR-WT + vehicle vs GR-WT + CORT**	**< 0.0001**
GR-WT +CORT	6	16.73	0.8961	**GR-WT + vehicle vs GR-2A + vehicle**	**0.0015**
GR-2A+vehicle	6	19.67	1.163	GR-2A + vehicle vs GR-2A + CORT	0.1961
GR-2A +CORT	6	16.99	0.9198	GR-WT + CORT vs GR-2A + CORT	0.9970

2B1 Apical spine diameter:

Two-way ANOVA Table	SS	DF	MS	F (DFn, DFd)	
Effect of GR-2A	0.01703	1	0.01703	F (1, 1264) = 1.303	
Effect of CORT	0.002304	1	0.002304	F (1, 1264) = 0.1761	
**GROUPS**	**N mice**	**Density**	**SEM**	**COMPARISONS**	**P value**
GR-WT+vehicle	6	0.3376	0.004258	**GR-WT + vehicle vs GR-WT + CORT**	**< 0.0001**
GR-WT +CORT	6	0.2819	0.004973	GR-WT + vehicle vs GR-2A + vehicle	0.9719
GR-2A+vehicle	6	0.3407	0.00525	**GR-2A + vehicle vs GR-2A + CORT**	**0.0181**
GR-2A +CORT	6	0.3629	0.006697	**GR-WT + CORT vs GR-2A + CORT**	**< 0.0001**

2B2 Apical spine length

Two-way ANOVA Table	SS	DF	MS	F (DFn, DFd)	
Effect of GR-2A	2.248	1	2.248	F (1, 1506) = 19.88	
Effect of CORT	3.488	1	3.488	F (1, 1506) = 30.84	
**GROUPS**	**N mice**	**Density**	**SEM**	**COMPARISONS**	**P value**
GR-WT+vehicle	6	0.8862	0.01575	**GR-WT + vehicle vs GR-WT + CORT**	**< 0.0001**
GR-WT +CORT	6	0.6937	0.01612	GR-WT + vehicle vs GR-2A + vehicle	0.9810
GR-2A+vehicle	6	0.8766	0.01679	GR-2A + vehicle vs GR-2A + CORT	0.963
GR-2A +CORT	6	0.8662	0.01659	**GR-WT + CORT vs GR-2A + CORT**	**< 0.0001**

2C Basal spine density:

Two-way ANOVA Table	SS	DF	MS	F (DFn, DFd)	
Effect of GR-2A	5.565	1	5.565	F (1, 16) = 2.238	
Effect of CORT	0.05512	1	0.05512	F (1, 16) = 0.02217	
**GROUPS**	**N mice**	**Density**	**SEM**	**COMPARISONS**	**P value**
GR-WT+vehicle	5	19.52	0.5614	GR-WT + vehicle vs GR-WT + CORT	0.9870
GR-WT +CORT	5	19.85	0.7604	GR-WT + vehicle vs GR-2A + vehicle	0.9236
GR-2A+vehicle	5	18.9	0.7391	GR-2A + vehicle vs GR-2A + CORT	0.9475
GR-2A +CORT	5	18.36	0.7413	GR-WT + CORT vs GR-2A + CORT	0.4636

2C1 Basal spine diameter:

Two-way ANOVA Table	SS	DF	MS	F (DFn, DFd)	
Effect of GR-2A	0.02025	1	0.02025	F (1, 1264) = 1.520	
Effect of CORT	0.0003127	1	0.0003127	F (1, 1264) = 0.02347	
**GROUPS**	**N mice**	**Density**	**SEM**	**COMPARISONS**	**P value**
GR-WT+vehicle	5	0.3953	0.006932	GR-WT + vehicle vs GR-WT + CORT	0.3865
GR-WT +CORT	5	0.3791	0.005443	GR-WT + vehicle vs GR-2A + vehicle	0.8198
GR-2A+vehicle	5	0.3865	0.006179	GR-2A + vehicle vs GR-2A + CORT	0.1881
GR-2A +CORT	5	0.4048	0.007377	**GR-WT + CORT vs GR-2A + CORT**	**0.0287**

2C2 Basal spine length:

Two-way ANOVA Table	SS	DF	MS	F (DFn, DFd)	
Effect of GR-2A	1.240	1	1.240	F (1, 1582) = 4.883	
Effect of CORT	0.07483	1	0.07483	F (1, 1582) = 0.2946	
**GROUPS**	**N mice**	**Density**	**SEM**	**COMPARISONS**	**P value**
GR-WT+vehicle	5	1.1820	0.028210	GR-WT + vehicle vs GR-WT + CORT	0.8106
GR-WT +CORT	5	1.2160	0.024800	GR-WT + vehicle vs GR-2A + vehicle	0.9953
GR-2A+vehicle	5	1.1730	0.023520	GR-2A + vehicle vs GR-2A + CORT	0.2781
GR-2A +CORT	5	1.1110	0.025140	**GR-WT + CORT vs GR-2A + CORT**	**0.0144**

Refers to Figure 3: Interaction of CORT with GR knockdown or TrkB deactivation. Density = number of spines scored on defined dendritic segment. SEM = standard error of the mean. N = number of mice per group. Two-way ANOVA, post-hoc Tukey's test showed that the GR_KD mutant or 1NaPP1 interfered with the effects of CORT when compared to the CTR shRNA groups. Values in bold are significant at *P* < 0.05.

3A Apical spine density:

Two-way ANOVA Table	SS	DF	MS	F (DFn, DFd)	
Effect of GR_KD	93.96	1	93.96	F (1, 16) = 15.15	
Effect of cort	63.90	1	63.90	F (1, 16) = 10.30	
**GROUPS**	**N mice**	**Density**	**SEM**	**COMPARISONS**	**P value**
CTR+vehicle	5	24.66	1.4780	**CTR + vehicle vs CTR + cort**	**0.0004**
CTR +cort	5	16.26	1.0970	**CTR + vehicle vs GR_KD + vehicle**	**0.0001**
GR_KD+vehicle	5	15.50	0.4108	CTR + cort vs GR_KD + cort	0.9892
GR_KD +cort	5	16.75	1.1860	GR_KD + vehicle vs GR_KD + cort	0.8564

3A1 Apical spine diameter:

Two-way ANOVA Table	SS	DF	MS	F (DFn, DFd)	
Effect of GR_KD	1.951	1	1.951	F (1, 889) = 279.9	
Effect of cort	0.1017	1	0.1017	F (1, 889) = 14.59	
**GROUPS**	**N mice**	**Diameter**	**SEM**	**COMPARISONS**	**P value**
CTR+vehicle	5	0.334300	0.004273	**CTR + vehicle vs CTR + cort**	**< 0.0001**
CTR +cort	5	0.294400	0.004186	**CTR + vehicle vs GR_KD + vehicle**	**< 0.0001**
GR_KD+vehicle	5	0.410600	0.006741	**CTR + cort vs GR_KD + cort**	**< 0.0001**
GR_KD +cort	5	0.407300	0.007142	GR_KD + vehicle vs GR_KD + cort	0.9791

3A2 Apical spine length

Two-way ANOVA Table	SS	DF	MS	F (DFn, DFd)	
Effect of GR_KD	15.05	1	15.05	F (1, 970) = 146.0	
Effect of cort	9.772	1	9.772	F (1, 970) = 94.83	
**GROUPS**	**N mice**	**Diameter**	**SEM**	**COMPARISONS**	**P value**
CTR+vehicle	5	0.970600	0.015470	**CTR + vehicle vs CTR + cort**	**< 0.0001**
CTR +cort	5	0.756600	0.016460	**CTR + vehicle vs GR_KD + vehicle**	**< 0.0001**
GR_KD+vehicle	5	1.207000	0.024220	**CTR + cort vs GR_KD + cort**	**< 0.0001**
GR_KD +cort	5	1.019000	0.025940	**GR_KD + vehicle vs GR_KD + cort**	**< 0.0001**

3B Basal spine density:

Two-way ANOVA Table	SS	DF	MS	F (DFn, DFd)	
Effect of GR_KD	23.98	1	23.98	F (1, 16) = 3.735	
Effect of cort	1.800	1	1.800	F (1, 16) = 0.2803	
**GROUPS**	**N mice**	**Density**	**SEM**	**COMPARISONS**	**P value**
CTR+vehicle	5	19.72	1.4350	CTR + vehicle vs CTR + cort	0.9712
CTR +cort	5	19.02	1.3250	CTR + vehicle vs GR_KD + vehicle	0.5005
GR_KD+vehicle	5	17.43	0.5049	CTR + cort vs GR_KD + cort	0.5736
GR_KD +cort	5	16.93	1.0330	GR_KD + vehicle vs GR_KD + cort	0.9891

3B1 Basal spine diameter:

Two-way ANOVA Table	SS	DF	MS	F (DFn, DFd)	
Effect of GR_KD	0.4147	1	0.4147	F (1, 1051) = 48.08	
Effect of cort	0.008678	1	0.008678	F (1, 1051) = 1.006	
**GROUPS**	**N mice**	**Diameter**	**SEM**	**COMPARISONS**	**P value**
CTR+vehicle	5	0.342400	0.005922	CTR + vehicle vs CTR + cort	0.8064
CTR +cort	5	0.335000	0.005476	**CTR + vehicle vs GR_KD + vehicle**	**< 0.0001**
GR_KD+vehicle	5	0.380500	0.005950	**CTR + cort vs GR_KD + cort**	**< 0.0001**
GR_KD +cort	5	0.376400	0.005414	GR_KD + vehicle vs GR_KD + cort	0.9570

3B2 Basal spine length:

Two-way ANOVA Table	SS	DF	MS	F (DFn, DFd)	
Effect of GR_KD	10.83	1	10.83	F (1, 1333) = 61.53	
Effect of cort	1.128	1	1.128	F (1, 1333) = 6.412	
**GROUPS**	**N mice**	**Diameter**	**SEM**	**COMPARISONS**	**P value**
CTR+vehicle	5	1.025000	0.015940	CTR + vehicle vs CTR + cort	0.8834
CTR +cort	5	1.002000	0.023950	**CTR + vehicle vs GR_KD + vehicle**	**< 0.0001**
GR_KD+vehicle	5	1.254000	0.031460	**CTR + cort vs GR_KD + cort**	**0.0003**
GR_KD +cort	5	1.154000	0.026450	**GR_KD + vehicle vs GR_KD + cort**	**0.0359**

3C Apical spine density:

Two-way ANOVA Table	SS	DF	MS	F (DFn, DFd)
Effect of 1NaPP1	9.591	1	9.591	F (1, 16) = 1.240
Effect of cort	82.22	1	82.22	F (1, 16) = 10.63

GROUPS	N mice	Density	SEM	COMPARISONS	P value
CTR+vehicle	5	26.62	1.9790	**CTR + vehicle vs CTR + cort**	**0.0006**
CTR +cort	5	17.71	1.1080	**CTR + vehicle vs 1NaPP1 + vehicle**	**0.0129**
1NaPP1+vehicle	5	20.38	0.5868	CTR + cort vs 1NaPP1 + cort	0.2388
1NaPP1 +cort	5	21.18	0.8371	1NaPP1 + vehicle vs 1NaPP1 + cort	0.9677

3C1 Apical spine diameter:

Two-way ANOVA Table	SS	DF	MS	F (DFn, DFd)
Effect of 1NaPP1	0.2561	1	0.2561	F (1, 1283) = 25.45
Effect of cort	0.6107	1	0.6107	F (1, 1283) = 60.68

GROUPS	N mice	Density	SEM	COMPARISONS	P value
CTR+vehicle	5	0.381900	0.007232	**CTR + vehicle vs CTR + cort**	**< 0.0001**
CTR +cort	5	0.2880	0.005194	**CTR + vehicle vs 1NaPP1 + vehicle**	**< 0.0001**
1NaPP1+vehicle	5	0.303400	0.005258	**CTR + cort vs 1NaPP1 + cort**	**0.0267**
1NaPP1 +cort	5	0.3099	0.004829	1NaPP1 + vehicle vs 1NaPP1 + cort	0.8300

3C2 Apical spine length

Two-way ANOVA Table	SS	DF	MS	F (DFn, DFd)
Effect of 1NaPP1	3.949	1	3.949	F (1, 1493) = 16.89
Effect of cort	10.23	1	10.23	F (1, 1493) = 43.76

GROUPS	N mice	Density	SEM	COMPARISONS	P value
CTR+vehicle	5	1.126000	0.023570	**CTR + vehicle vs CTR + cort**	**< 0.0001**
CTR +cort	5	0.8471	0.023470	CTR + vehicle vs 1NaPP1 + vehicle	0.9988
1NaPP1+vehicle	5	1.121000	0.024680	**CTR + cort vs 1NaPP1 + cort**	**< 0.0001**
1NaPP1 +cort	5	1.0620	0.025380	1NaPP1 + vehicle vs 1NaPP1 + cort	0.2984

3D Basal spine density:

Two-way ANOVA Table	SS	DF	MS	F (DFn, DFd)
Effect of 1NaPP1	0.8688	1	0.8688	F (1, 16) = 0.06774
Effect of cort	0.03606	1	0.03606	F (1, 16) = 0.002812

GROUPS	N mice	Density	SEM	COMPARISONS	P value
CTR+vehicle	5	20.36651	0.743836	CTR + vehicle vs CTR + cort	0.9995
CTR +cort	5	20.62348	1.279985	CTR + vehicle vs 1NaPP1 + vehicle	0.9866
1NaPP1+vehicle	5	21.12525	1.949315	CTR + cort vs 1NaPP1 + cort	> 0.9999
1NaPP1 +cort	5	20.69843	2.066011	1NaPP1 + vehicle vs 1NaPP1 + cort	0.9975

3D1 Basal spine diameter:

Two-way ANOVA Table	SS	DF	MS	F (DFn, DFd)
Effect of 1NaPP1	5.673e-006	1	5.673e-006	F (1, 1023) = 0.0007391
Effect of cort	0.0007720	1	0.0007720	F (1, 1023) = 0.1006

GROUPS	N mice	Density	SEM	COMPARISONS	P value
CTR+vehicle	5	0.330800	0.005837	CTR + vehicle vs CTR + cort	0.5468
CTR +cort	5	0.34130	0.005460	CTR + vehicle vs 1NaPP1 + vehicle	0.6888
1NaPP1+vehicle	5	0.339700	0.004645	CTR + cort vs 1NaPP1 + cort	0.6622
1NaPP1 +cort	5	0.33270	0.005904	1NaPP1 + vehicle vs 1NaPP1 + cort	0.7990

3D2 Basal spine length:

Two-way ANOVA Table	SS	DF	MS	F (DFn, DFd)
Effect of 1NaPP1	10.23	1	10.23	F (1, 1327) = 46.61
Effect of cort	0.1017	1	0.1017	F (1, 1327) = 0.4635

GROUPS	N mice	Density	SEM	COMPARISONS	P value
CTR+vehicle	5	1.050000	0.024350	CTR + vehicle vs CTR + cort	0.8835
CTR +cort	5	1.07700	0.024340	**CTR + vehicle vs 1NaPP1 + vehicle**	**< 0.0001**
1NaPP1+vehicle	5	1.235000	0.026150	**CTR + cort vs 1NaPP1 + cort**	**< 0.0001**
1NaPP1 +cort	5	1.24300	0.027500	1NaPP1 + vehicle vs 1NaPP1 + cort	0.9961

Refers to Figure 4A,B : Expression of DUSP1 as a function of p-GR in the mouse cortex. N = number of mice/ group. Unpaired t-test showed significant difference of DUSP1 and p-GR between vehicle and CORT groups. Values in bold are significant at *P* < 0.05.

Unpaired t-test	t, df	R squared	Mean ± SEM relative to naïve		P value	N
**Effect of CORT on DUSP1**	t=4.238 df=16	0.4944	0.6 ± 0.07234		**0.0006**	18 (9 vehicle, 9 CORT))
**Effect of CORT on p-GR**	t=6.47 df=16	0.7022	0.4303 ± 0.0449		**< 0.0001**	18 (9 vehicle, 9 CORT))
**Correlation**	**Pearson r**	**R squared**	**DFn, DFd**	**F value**	**P value**	**N**
**DUSP1 levels vs. p-GR levels**	0.8362	0.6993	1.000, 16.00	37.2	**<0.0001**	18 (9 vehicle, 9 CORT)

Refers to Figure 4D: Effect of DUSP1 knockdown on PHF1 phosphorylation and interaction with CORT in the mouse cortex. SEM = standard error of the mean. N = number of mice per group. Two-way ANOVA, post-hoc Tukey's test showed that DUSP1_KD interfered with the effects of CORT when compared to the CTR groups. Values in bold face type are significant at *P* < 0.05.

4D effect of shRNA DUSP1

Two-way ANOVA Table	SS	DF	MS	F (DFn, DFd)	
Effect of CORT	52.86	1	52.86	F (1, 20) = 6.660	
Effect of DUSP1 knockdown (GFP)	199.2	1	199.2	F (1, 20) = 25.10	
**GROUPS**	**N mice**	**Diameter**	**SEM**	**COMPARISONS**	**P value**
GFP negative cells (CTR) +vehicle	5	2.730718	0.6842529	**CTR + vehicle vs CTR + cort**	**0.0210**
GFP negative cels (CTR) +cort	7	8.025554	1.051283	**CTR + vehicle vs DUSP1_KD+ vehicle**	**0.001**
GFP cells +vehicle	5	10.85933	1.613658	CTR + cort vs DUSP1_KD + cort	0.1170
GFP cells +cort	7	11.5849	1.100471	DUSP1_KD + vehicle vs DUSP1_KD + cort	0.9708

4D1 effect of shRNA CONTROL

Two-way ANOVA Table	SS	DF	MS	F (DFn, DFd)	
Effect of CORT	116.6	1	116.6	F (1, 24) = 26.70	
Effect of shRNA CONTROL (GFP)	6.212	1	6.212	F (1, 24) = 1.423	
**GROUPS**	**N mice**	**Diameter**	**SEM**	**COMPARISONS**	**P value**
GFP negative cells (CTR) +vehicle	7	2.019939	0.3640538	**CTR + vehicle vs CTR + cort**	**0.0020**
GFP negative cells (CTR) +CORT	6	6.820	1.074688	CTR + vehicle vs shRNA CONTROL + vehicle	0.9960
GFP cells +vehicle	7	1.771054	0.4641698	CTR + cort vs shRNA CONTROL + cort	0.4775
GFP cells +CORT	8	5.175	0.9958396	**shRNA CONTROL + vehicle vs shRNA CONTROL + cort**	**0.0212**

Refers to Figure 4E-E1: Interaction of DUSP1 knockdown and CORT on spine density in the cortex. Density = number of spines scored on defined dendritic segment. SEM = standard error of the mean. N = number of mice per group. Unpaired t-test showed significant difference of spine density between vehicle and CORT groups. Values in bold face type are significant at *P* < 0.05.

Unpaired t-test	t, df	R squared	Spine density Mean ± SEM	P value	N mice
**Apical dendrites :**					
**Effect of CORT on shRNA DUSP1**	**t=5.492 df=10**	**0.7510**		**0.0003**	
**Effect of CORT on shRNA CONTROL**	**t=2.472 df=8**	**0.4331**		**0.0386**	
shRNA DUSP1 + vehicle			30.68 ± 1.137		6 vehicle
shRNA DUSP1 + CORT			21.30 ± 1.274		6 CORT
shRNA CONTROL + vehicle			24.97 ± 1.849		5 vehicle
shNRA CONTROL + CORT			17.68 ± 2.300		5 CORT
**Basal dendrites :**					
**Effect of CORT on shRNA DUSP1**	**t=4.201 df=12**	**0.5953**		**0.0012**	
Effect of CORT on shRNA CONTROL	t=0.5124 df=8	0.03178		0.6222	
shRNA DUSP1 + vehicle			28.35 ± 1.117		7 vehicle
shRNA DUSP1 + CORT			22.11 ± 0.9797		7 CORT
shRNA CONTROL + vehicle			19.06 ± 1.205		5 vehicle
shNRA CONTROL + CORT			18.21 ± 1.115		5 CORT

Refers to Figure 5B: Effect of DUSP1 overexpression on PHF1 phosphorylation in the mouse cortex. Mean +- SEM = standard error of the mean. Two-way ANOVA, post-hoc Tukey's test showed that the DUSP1 overexpression interfered with the effects of CORT on PHF1 phosphorylation. Values in bold are significant at *P* < 0.05.

Two-way ANOVA Table	SS	DF	MS	F (DFn, DFd)	
DUSP1 overexpression	87.33	1	87.33	F (1, 20) = 52.67	
Corticosterone	78.74	1	78.74	F (1, 20) = 47.49	
**GROUPS**	**N mice**	**Density**	**SEM**	**COMPARISONS**	**P value**
GFP + vehicle	6	0.73530	0.11450	**GFP + vehicle vs GFP + CORT**	**< 0.0001**
GFP + CORT	4	7.875	1.5940	GFP + vehicle vs DUSP1 + vehicle	0.8924
DUSP1 + vehicle	6	0.20830	0.02676	**GFP + CORT vs DUSP1 + CORT**	**< 0.0001**
DUSP1 + CORT	8	0.537	0.2007	DUSP1 + vehicle vs DUSP1 + CORT	0.9642

Refers to Figure 5F: Effect of DUSP1-RFP minigene on spine density in the mouse cortex. Density = number of spines scored on defined dendritic segments. SEM = standard error of the mean. Unpaired t-test showed no significant difference of spine density between vehicle and CORT groups. Values in bold are significant at *P* < 0.05.

Unpaired t-test	t, df	R squared	Spine density (Mean ± SEM)	P value	N mice
Apical dendrites :					
Effect of CORT on cells with DUSP1 minigene	t=0.03234 df=8	0.0001307		0.9750	
Minigene + vehicle			24.43 ± 0.4417		5 vehicle
Minigene + CORT			24.34 ± 2.591		5 CORT
Basal dendrites :					
Effect of CORT on cells with DUSP1 minigene	t=1.001 df=6	0.1431		0.3555	
Minigene + vehicle			20.28 ± 1.551		4 vehicle
Minigene + CORT			18.64 ± 0.5127		4 CORT

Refers to Figure 6B : Expression of DUSP1 protein in human cohort 1. N = number of patients/ group. Unpaired t-test showed significant difference of DUSP1 expression between CTR and AD groups. Values in bold face type are significant at *P* < 0.05.

Unpaired *t*-test	CTL (N)	AD (N)	P value	T value
DUSP1 protein	8.488 ± 1.688 *(10)*	4.693 ± 0.5469 *(15)*	0.0202	2.495

Refers to Figure 6C : Expression of DUSP1 protein in human cohort 2. N = number of patients/ group. Unpaired t-test showed significant difference of DUSP1 expression between CTR and SEVERE groups. Values in bold face type are significant at *P* < 0.05.

Unpaired t-test	t, df	R squared	DUSP1 (Mean ± SEM)	P value	N patient
CTR vs MILD	t=1.007 df=20	0.04828		0.3259	22
**CTR vs SEVERE**	**t=2.419 df=39**	**0.1304**		**0.0203**	**41**
MILD vs SEVERE	t=0.6338 df=27	0.01466		0.5315	29
CTR			0.5796 ± 0.05299		17
MILD			0.4768 ± 0.04557		5
SEVERE			0.4139 ± 0.04377		24

Refers to Figure 6D: Expression of PSD95 in human cohort 2 and correlation with DUSP1. N = number of patients/ group. Unpaired t-test showed significant difference of PSD95 expression between CTR and SEVERE groups. Values in bold face type are significant at $P < 0.05$.

Unpaired t-test	t, df	R squared	PSD95 (Mean ± SEM)	P value	N patient
CTR vs MILD	t=0.8931 df=20	0.03835		0.3824	22
**CTR vs SEVERE**	**t=2.795 df=39**	**0.1669**		**0.008**	**41**
MILD vs SEVERE	t=0.9546 df=27	0.03265		0.3482	29
CTR			0.3605 ± 0.07828		17
MILD			0.2243 ± 0.08067		5
SEVERE			0.1445 ± 0.03435		24

Correlation	Pearson r	R squared	DFn, DFd	F value	P value	N
**DUSP1 levels vs. PSD95 levels**	0.5393	0.2909	1.000, 44.00	17.64	**0.0001**	46
